# Criticality Assessment of the Life Cycle of Passenger Vehicles Produced in China

**DOI:** 10.1007/s43615-021-00012-5

**Published:** 2021-02-23

**Authors:** Xin Sun, Vanessa Bach, Matthias Finkbeiner, Jianxin Yang

**Affiliations:** 1 China Automotive Technology and Research Center Co., Ltd, No. 68, East Xianfeng Road, Dongli District, Tianjin, 300300 China; 2 State Key Laboratory of Urban and Regional Ecology, Research Center for Eco-Environmental Sciences, Chinese Academy of Sciences, No.18 Shuangqing road, Haidian District, Beijing, 100085 China; 3 College of Resources and Environment, University of Chinese Academy of Sciences, No. 80 East Zhongguancun Road, Haidian District, Beijing, 100190 China; 4 Technische Universität Berlin, Chair of Sustainable Enginnering, Straße des 17. Juni 135, 10623 Berlin, Germany

**Keywords:** Criticality, Resources, ESSENZ, Life cycle assessment, Environmental impacts, E-mobility

## Abstract

China is globally the largest and a rapidly growing market for electric vehicles. The aim of the paper is to determine challenges related to criticality and environmental impacts of battery electric vehicles and internal combustion engine vehicles, focusing not only on a global but also the Chinese perspective, applying the ESSENZ method, which covers a unique approach to determine criticality aspects as well as integrating life cycle assessment results. Real industry data for vehicles and batteries produced in China was collected. Further, for the criticality assessment, Chinese import patterns are analyzed. The results show that the battery electric vehicle has similar and partly increased environmental impacts compared with the internal combustion engine vehicle. For both, the vehicle cycle contributes to a large proportion in all the environmental impact categories except for global warming. Further, battery electric vehicles show a higher criticality than internal combustion engine vehicles, with tantalum, lithium, and cobalt playing essential roles. In addition, the Chinese-specific results show a lower criticality compared to the global assessment for the considered categories trade barriers and political stability, while again tantalum crude oil and cobalt have high potential supply disruptions. Concluding, battery electric vehicles still face challenges regarding their environmental as well as criticality performance from the whole supply chain both in China and worldwide. One reason is the replacement of the lithium-ion power battery. By enhancing its quality and establishing battery recycling, the impacts of battery electric vehicle would decrease.

**Supplementary Information:**

The online version contains supplementary material available at 10.1007/s43615-021-00012-5.

## Introduction

In 2018, the global transportation sector accounted for 36% of the world’s total final energy consumption. Within the transportation sector, passenger vehicles account for 58% of the global transportation sector and more than 20% of the world’s total final energy consumption and carbon dioxide (CO_2_) emission [1, 2].

In recent years, to achieve energy conservation, resource security, and a decrease in environmental pollution in the transportation sector, electric vehicles (EVs), including battery electric vehicles (BEVs) and plug-in hybrid electric vehicles (PHEVs), have been promoted by many countries [3–9].

Globally, sales of EVs topped 2.1 million globally in 2019, surpassing 2018 to boost the stock to 7.2 million EVs [10]. China remained the world’s largest EV market with 2.6 million EV stock, accounting for 46.5% of global EV sales in 2018 [10]. In China, EV sales have experienced rapid growth in recent years motivated by numerous policies and programs by the Chinese government. From 2009 to 2019, annual passenger EV sales grew from 0.5 thousand to 1.1 million, with an average annual growth rate of 136% [10]. Out of a total 3.4 million EVs sold in the last 10 years, 74% of them were BEVs [10].

The global EV stock consumed an estimated 80 terawatt-hours (TWh) of electricity in 2019, of which China accounted for the most proportion. Meanwhile, the global EV fleet in 2019 emitted about 51 million tons of carbon-dioxide equivalent (Mt CO2-eq) on a well-to-wheel basis, about half the amount that from internal combustion engine vehicles (ICEVs) fleet [10].

Life cycle assessment (LCA) has been proposed to compile and evaluate inputs, outputs, and the potential environmental impact of a product system throughout its life cycle [11]. Until now, several LCA studies have shown that BEVs could substantially reduce energy consumption and environmental impacts compared with ICEVs from fuel cycle (well to wheels) in China for the same transportation service provided, especially with clean energy increasingly dominating the electricity mix [12–15]. In addition, several studies have also found that BEVs hold significant advantages in life cycle CO_2_, SO_2_, VOCs, CO, NO_x_, and PM_2.5_ emissions’ reduction compared with ICEVs [16–18]. However, for the whole life cycle, including the vehicle cycle and fuel cycle, several studies have presented that BEVs have larger abiotic depletion potential, eutrophication potential, and other environmental impacts than ICEVs [16, 19–21].

In addition, the use of abiotic resource over the life cycle of BEVs as well as ICEVs has been analyzed in several publications (e.g. [22–25]), coming to the conclusion that BEVs have a lower impact on fossil resource use than ICEVs due to decreased use of fossil fuels during the use phase. For mineral resource use, this picture shifts, because BEVs usually have a higher resource use compared to ICEVs as shown for example by Cimprich et al. [26], Hawkins et al. [27], and Helmers et al. [28]. The same results arise when looking at criticality: Due to needs of critical resource for battery production, the overall criticality of EVs is usually higher compared to ICEVs. However, so far only few studies exist [26, 29, 30] analyzing critical resources. And so far, none with the viewpoint of Chi((nese BEVs and ICEVs production [31]. Criticality refers to the “risk” of supply disruption (or “supply risk”) together with potential (socio-economic) impacts of supply disruption (“vulnerability to supply disruption”), sometimes including other dimensions such as environmental and social aspects [32].

Thus, identified challenges about the use of abiotic resources related to battery production of BEVs still exist. This includes the comparison of criticality aspects for ICEVs and BEVs as well as the reduction potential in resource use, energy consumption, and environmental impacts of BEVs compared to ICEVs from a Chinese perspective (a paper outline is shown in Fig. S1 in the supplementary information).

The objective of this study is to determine challenges related to criticality and environmental impacts of ICEV and BEV. Even though some studies have addressed criticality issues from a Chinese (macro level) perspective [33], so far, a product (micro level) perspective has not been considered. The difference between a macro level and a micro level perspective is the following: whereas on a macro level, used raw materials of a region or sector are considered applying import data or material flow analysis, on a micro level perspective, only the materials within the specific product (system) are evaluated applying method like LCA. Thus, product criticality approaches are applied to determine criticality hotspots of products [32, 34, 35]. Further, for the first time, Chinese-specific characterization factors are derived in applied for the ESSENZ method. Additionally, specific Chinese inventory data is used within this paper an environmental assessment (applying LCA) as well as a comprehensive criticality assessment with focus on the Chinese raw material situation applying the ESSENZ approach is carried out. Compared to existing literature, the following aspects are new to this study: use of Chinese-specific inventory data for the environmental as well as criticality assessment (existing studies often rely on secondary literature as well as input-output tables), deriving criticality results for a variety of aspects (existing papers focus on political stability, concentration, and policy perception only) from a global as well as Chinese product perspective (existing studies often use a European product level approach or a Chinse macro level approach).

Thus, based on the identified research gaps, the following two research questions are answered in this paper:

1. What are the environmental impacts of BEV compared to ICEV considering Chinese inventory data?
2. Does the criticality of raw materials for the new technology BEV is also an important issue from a Chinese perspective?

By considering not only environmental but also criticality aspects, trade-offs between these two dimensions are shown, which helps to derive adequate recommendations for decision makers. This paper therefore contributes to the discussion on the topic of e-mobility by providing a new perspective. The intended audience is therefore researchers working on the topic e-mobility and developing methods for criticality as well as decision-makers implementing new strategies related to mobility.

An LCA according to ISO 14040 series standards [36] of the ICEV and the BEV is carried out. Further, criticality aspects are considered by applying the integrated method to assess resource efficiency (herein referred to as ESSENZ) and adapting it to allow for a Chinese perspective considering the unique Chinese raw material situation [37], because China is one of the main producers of many materials used in ICEVs and BEV. Often, these materials are considered to be critical from a European and/or North American perspective, but might not be critical from a Chinese perspective. The ESSENZ method has been applied, because it was just recently identified as one of the most appropriate approaches available to address criticality aspects within product assessments within the UNEP/SETAC task force for mineral resources [34, 35, 38]. For transparency, it is highlighted here that the only other existing approach to determine criticality on a product level also got a good rating. However, as some of the authors of the paper developed the ESSENZ approach and therefore know its limitations as well as how to apply it by heart, it seems the obvious choice. The ESSENZ method evaluates abiotic resource use—mainly metals and fossil raw materials—in the context of sustainable development focusing on criticality assessment as well as evaluating environmental impacts. The applied approach for the criticality assessment is a screening approach only, meaning that the results should be interpreted as “potential criticality issues.” The dimensions and categories of ESSENZ are shown in Table 1. For details, please see the original publications [39, 40] and supplementary information.

**Table 1:** Overview on dimensions and categories of ESSENZ

Sustainability dimension	Dimension of ESSENZ	Categories
Economy	Criticality (potential supply disruption)	Concentration of production, reserves, and company concentration
		Feasibility of exploration projects
		Political stability
		Occurrence of co-product
		Mining capacity
		Primary material use
		Demand growth
		Price fluctuations
		Trade barriers
	Physical availability	Abiotic resource depletion
Environment	Environmental impacts	Climate change
		Acidification
		Eutrophication
		Smog
Society	Societal acceptance	Compliance with social standards
		Compliance with environmental standards

### Background—ESSENZ Method

The ESSENZ method not only focusses on criticality assessment but also addresses environmental impacts of resource use along the supply chain of the considered product system. For determining environmental impacts, the life cycle impact assessment method CML-IA [41] is applied for the impact categories climate change, acidification, eutrophication, and smog. The CML-IA method was chosen to be applied in ESSENZ, because it is one of the mostly used approaches in Europe, but does not focus on European distribution and fate, therefore making it applicable for assessing supply stage steps and life cycles which take part in different parts of the world [39, 42]. The criticality dimension is assessed by considering the physical (long term) availability of resources based on [43–45] and socio-economic (medium term) availability (also called criticality) of resources. The criticality is determined by considering potential supply disruptions along the supply chain through the following eleven categories:

- Concentration of production, reserves, and company concentration: High concentrations refer to few countries and/or companies that mine and trade resources, which can lead to potential supply disruptions, measured by the Herfindahl-Hirschman Index (HHI) [46].
- Feasibility of exploration projects: Political and societal factors (taxation, environmental regulations, administration of regulations, or infrastructure) influence the opening; e.g., mine development is delayed or canceled, measured by the policy potential index (PPI) [47].
- Political stability: Governance instability (e.g., corruption) interrupts production, measured by the worldwide governance indicators (WGI) [48].
- Occurrence as co-product: restriction of companion metals, when host metals are not being mined anymore, measured by percentage of production as companion metal [49].
- Mining capacity: Remaining time to extract resources in already developed mines worldwide considering current conditions (e.g., technological and economic feasibility) is too short, measured by the reserve-to-annual-production ratio.
- Primary material use: Secondary materials are only used to a certain extent; thus, more primary materials need to be extracted, measured by the percentage of new material content [50].
- Demand growth: If the demand increases significantly, current production might not be able to keep up, measured by the percentage of annual growth based on past.
- Price fluctuations: Significant unexpected price fluctuations can lead to higher prices of a resource that a company might no longer afford, measured by the volatility indicator [51].
- Trade barriers: barriers to material trade (e.g., due to export duties), measured by the enabling trade index [52].

For determining the global ESSENZ characterization factors (CFs), global production data by United States Geological Survey [53] and British Geological Survey [54] is used. The results are determined separately for each category.

The characterization factor of ESSENZ for each resource *i* in category *c* is determined based on the ecological scarcity approach [55, 56], carrying out the following steps: (i) indicator result of a specific resource for the considered category is calculated; (ii) the indicator result is set in relation with the category specific target value; (iii) distance-to-target values are normalized; and (iv) scaling of final characterization factors. To carry out step (ii), for each category, individual target values based on expert judgments and stakeholder surveys have been determined (details are explained in Bach et al. [39]). The target value is the point where resources have no potential supply disruption. By setting the indicator result in relation to the target value, it can be determined if a potential supply disruption occurs. If the calculated value is above 1, potential supply disruptions are likely to occur. If the value is below 1, the risk for potential supply disruption is low. Distance-to-target values below 1 are set to zero to avoid a distortion of the overall results. The normalization factor applied in step (iii) is the globally produced amount of the considered resource. By dividing the distance-to-target result by the normalization factor, effects of the overall amount of the resource currently produced are taken into account. The final characterization factors are multiplied with the inventory data of the considered product system.

The final CFs applied in the case study of this paper can be found here: https://www.see.tu-berlin.de/menue/forschung/daten_tools /essenz/parameter/en/.

## Methods

### Description of the Product System and System Boundaries

The functional unit in this study is one passenger vehicle driven over its lifetime of 150,000 km. The system boundary covers the life cycle stages of a vehicle, including the vehicle cycle (including the manufacturing of vehicles) and the fuel cycle (including the use phase). The vehicle cycle covers the acquisition of raw materials, the preparation of materials, Li-ion power battery (LIB) manufacturing, the vehicle production, and the maintenance. The fuel cycle covers the extraction, refining, transportation and distribution of fuels (well to pump, WTP), and vehicle use (pump to wheels, PTW). Regarding the fuel cycle, the system boundary of ICEV includes oil extraction, transportation, refining/processing and transportation of fuel, and vehicle use, while those of BEV include electricity generation (including fossil fuel, hydropower, wind power, solar power, and nuclear power), electricity transmission and distribution, BEV charge, and vehicle use [15]. The manufacture of the capital equipment, such as onsite structures, machinery, and other infrastructure, was not considered.

The average A size class ICEV and BEV in China were selected as the reference passenger vehicles. The cumulative sales weighted average method is used to calculate the average technical data of A size class ICEV and BEV. In accordance with the formulas below, the cumulative sales weighted average (SWA) test fuel consumption (F_T_), SWA test exhaust gas emission (E_T_), SWA vehicle weight (W_V_), and SWA battery weight (W_B_) and of passenger vehicles are calculated and shown in Table 2.

1 $$ {F}_T=\frac{\sum \left({F}_{ij}\times {S}_{ij}\right)}{\sum {S}_{ij}} $$

2 $$ {E}_T=\frac{\sum \left({E}_{ij}\times {S}_{ij}\right)}{\sum {S}_{ij}} $$

3 $$ {W}_v=\frac{\sum \left({WV}_{ij}\times {S}_{ij}\right)}{\sum {S}_{ij}} $$

4 $$ {W}_B=\frac{\sum \left({WB}_{ij}\times {S}_{ij}\right)}{\sum {S}_{ij}} $$

where *F*_ij_ is the test fuel consumption of passenger vehicle i in year j; *E*_ij_ is the test exhaust gas emission of passenger vehicle i in year j; *WV*_ij_ is the vehicle weight of passenger vehicle i in year j; *WB*_ij_ is the battery weight of passenger vehicle i in year j; and *S*_ij_ is the cumulative sales volume of passenger vehicle i in year j.

**Table 2:** Cumulative sales weighted average (SWA) data for passenger vehicles from 2011 to 2018

Passenger vehicle type	SWA fuel consumption (F_T_)	SWA vehicle weight (W_V_) (kg)	SWA battery weight (W_B_) (kg)	SWA battery capacity (C) (kWh)	SWA CO (g/km)	SWA NO_x_ (g/km)	SWA PM (g/km)	SWA CH_4_ (g/km)	Cumulative sales from 2011 to 2018
ICEV	6.9 (L/100 km)	1355.6	--	--	0.430	0.029	0.005	0.014	67,102,574
BEV	14.9 (kWh/100 km)	1672.6	398.0	44.6	--	--	--	--	352,331

The cumulative sales weighted average data, laboratory test fuel consumption, exhaust gas emission, vehicle weight, battery capacity, and battery weight, which come from China Automotive Technology and Research Center Co., Ltd, cover all the passenger vehicles sold in China from 2011 to 2018.

The ESSENZ method is applied to determine environmental as well as criticality aspects of the considered system. For the determination of environmental impacts, CML-IA baseline V3.02 impact assessment method [41] is applied for the impact categories climate change (global warming potential, GWP), acidification (acidification potential, AP), eutrophication (eutrophication potential, EP), and smog (photochemical oxidant creation potential, POCP). These categories were chosen, because these are the categories most often applied in most LCA case studies [42, 57, 58]. This allows for an easy communication. SimaPro 8 software (PRé Sustainability, Netherlands) was used as a support tool to establish the LCA model and perform the environmental impact assessment. Criticality results are determined for the 11 categories (presented in the background) —applying the global ESSENZ approach as well as an adapted approach to take into account Chinese production situations (for details see Section 2.3).

### Life Cycle Inventory

Life cycle inventory (LCI) is the inventory of input/output data with regard to the vehicle system being studied [11]. LCI data include background data and foreground data. The background data, including the LCI data in the acquisition of raw materials, the preparation of materials, and the production of energy and fuels, were based on the China Automotive Life Cycle Database 2018 (CALCD) [59–61], which is a local Chinese process-based LCI database developed by the China Automotive Technology and Research Center Co., Ltd. Foreground data consist of the LCI data in the vehicle cycle and fuel cycle. For the LCI data in the vehicle cycle, the vehicle material composition data from the onsite surveys of 10 models (with total sales of more than 2 billion until 2018) in six Chinese leading automotive corporations in 2018 were used. The energy consumption data in the vehicle production process were based on onsite investigations in 24 Chinese automotive factories from 2015 to 2018. The material composition and energy consumption data of LIBs were collected from the onsite investigations in two Chinese leading LIB suppliers (world’s top three) and two leading cathode material producer (world’s top five) from 2017 to 2019 in China [62].

Table 3 shows the cumulative sales weighted average weights of the vehicle components of passenger vehicles. In this study, it is assumed that the BEV is powered by the lithium nickel cobalt manganese oxide (LiNixCoyMnzO2, NCM 622) battery, which has been the most commonly used in electric passenger vehicles in China (CATARC and BIT, 2019). The results without considering the replacement if LIBs are presented in the supplementary material (see Chapter 4).

**Table 3:** Cumulative sales weighted average component weights for per ICEV and BEVs

	ICEV	BEV
Other components (kg)	1257.3	1240.6
Lead-acid battery (kg)	16.6	14.2
Li-ion power battery (kg)	0.0	352.0
Tyres (kg)	48.3	49.6
Fluids (kg)	33.3	16.2
Total vehicle weight (kg)	1355.6	1,672.6

**Figure 1:**
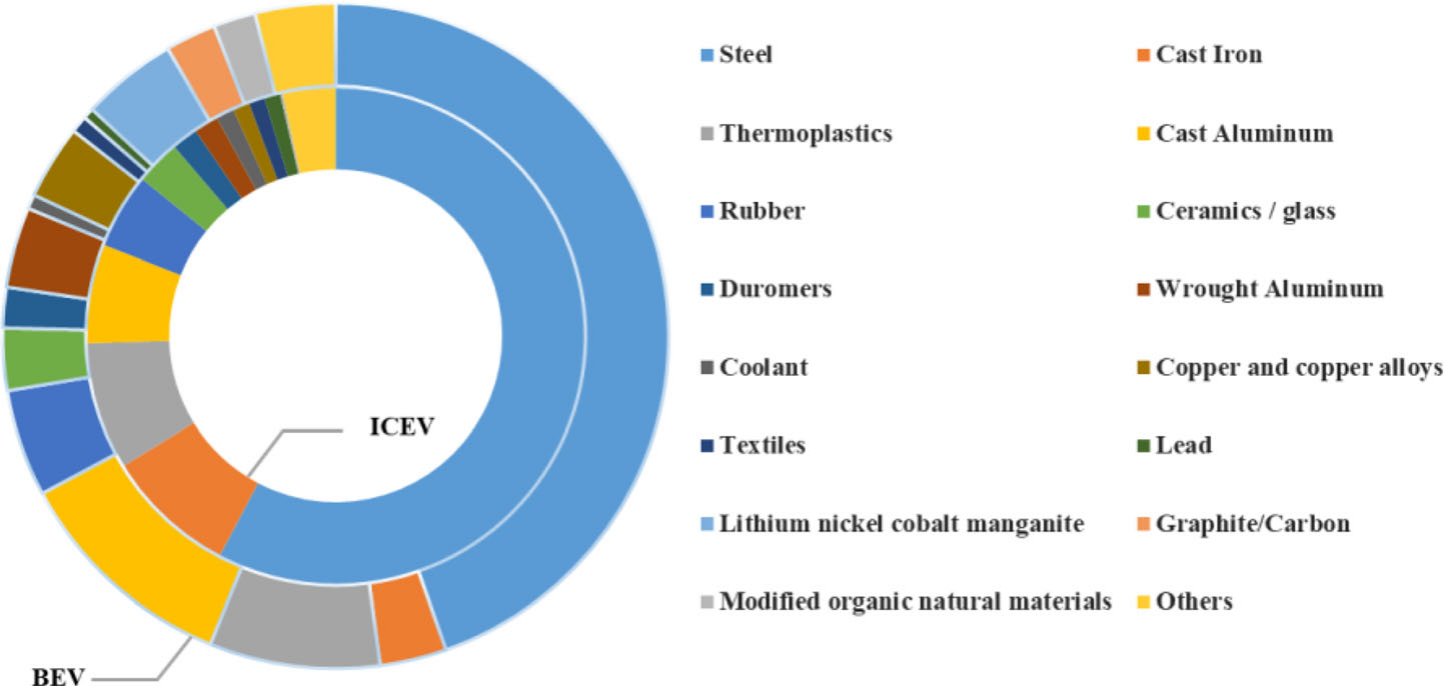
Cumulative sales weighted average material composition for ICEVs and BEVs

Figure 1 shows the cumulative sales weighted average material composition for ICEVs and BEVs. Steel contributes the largest proportion (around 50%) for both ICEVs and BEVs, followed by cast aluminum (around 10%), thermoplastics (8%), and cast iron (around 5%). The BEV uses less steel, cast iron, and fluids, but more wrought aluminum, cast aluminum, magnesium, copper, and thermoplastics. NCM, graphite, and electrolyte are the specific materials for BEV, which are used by the LIBs. Platinum/rhodium are the specific materials for ICEV, which are used by the three-way catalyst. Detailed material compositions for ICEVs and BEVs are presented in Table S1 in the Supplementary Information.

Table 4 shows the replacement information during the vehicle use phase (150,000 km). From enterprise investigation, this study considered the replacement of tyre, fluid, and lead-acid battery for both the ICEV and BEV. Besides, it assumed that the whole LIB pack was replaced once in the whole BEV’s lifetime [63].

**Table 4:** Tyre, fluids, and batteries’ replacement times during the vehicle use phase

	ICEV	BEV
Lead-acid battery	2	2
Li-ion power battery	0	1
Tyres	2	2
Lubricants	29	29
Brake fluid	2	2
Coolant	2	2
Refrigerant	1	1
Washing water	14	14

Based on onsite measurements, the energy consumption of the vehicle production is 3599 MJ and 2302 MJ for the ICEV and the BEV, respectively. The energy consumption of the LIB pack production for the BEV sums up to 6975 MJ.

In the fuel cycle, the cumulative sales weighted average test fuel consumption is 6.9 L/100 km of gasoline for the ICEV and 14.9 kWh/100 km of electricity for the BEV. Chinese electricity grid mix includes 71.0% of thermal (64.7% of coal, 3.2% of gas and 3.2% of oil), 18.6% of hydro power, 4.7% of wind, 1.8% of solar, and 3.9% of nuclear power in 2017 [64]. Besides, the cumulative sales weighted average exhaust gases, including 0.430 g/km CO, 0.029 g/km NO_x_, 0.005 g/km PM, 0.014 CH_4_, and 162.6 g/km CO_2_, are emitted in the PTW stage of the ICEV (see Table 1 in the Supplementary Information).

### Application of ESSENZ Method and Adaptation to Chinese Situation

In a first step, the original ESSENZ CFs as described in Section 1.1 are applied. These factors are based on the global production mix. In a second step, the CFs were adapted to reflect Chinese production patterns.

ESSENZ is based on global production mix based on data by United States Geological Survey [53] and British Geological Survey [54]. For the adaptation to the Chinese situation, the Chinese import mix based on data by General Administration of Customs of the People’s Republic of China [65] was used. Based on the import mix and China’s own production, the import/production shares are determined. Next, the indicator results for the import mix are calculated according to the ESSENZ approach (please see Bach, Berger [39] for further details as well as the supplementary information). To minimize distorting the results by subjective perception of risk, the same target values (as used in ESSENZ, which were derived based on a survey of stakeholders in Europe) were used. Deriving a global set of target values would have been favorable, but was not possible in the scope of the project.

Domestic aspects restricting the supply of raw materials, e.g., local policies, were not taken into account, but might lead to supply restrictions. Thus, it was assumed that resources produced in China do not face any supply restrictions. For the share produced in China, the supply disruption is set to zero similarly to an approach introduced by Helbig et al. [66].

The following steps are equal to the steps carried out in ESSENZ (see steps (ii) to (iv) in the background). The adapted characterization factors are only determined for the abiotic resources used in the conducted case study.

As shown in Bach et al. [39], only few of the categories of the dimension criticality are actually country specific. Price fluctuations for example is one of the categories which is determined based on global market prices and therefore not specific for a country. Thus, for this case study, only the categories trade barriers and political stability are analyzed in detail, because they are highly influenced by the country’s producing/mining the material (e.g., on a global level, many abiotic raw materials are flagged as critical in the considered categories due to high production amounts in China) and can be compared to the global results.

## Results

In the following, the results of the environmental impacts (Section 3.1) as well as the criticality assessment (Section 3.2) are presented and explained in detail.

### Environmental Impacts

**Figure 2:**
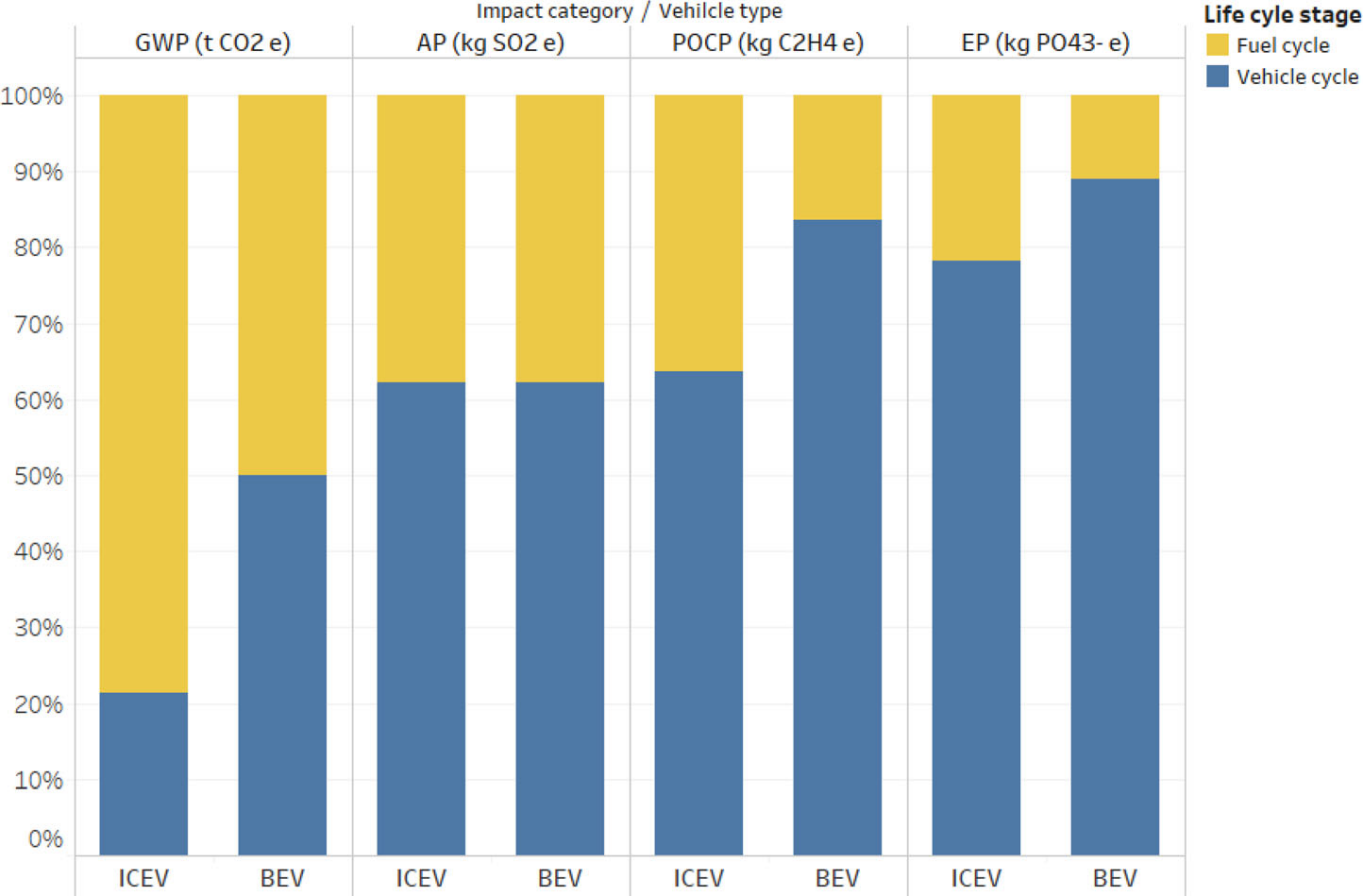
Life cycle environmental impacts comparison for the ICEV and BEV

The results of the two considered passenger vehicles (the ICEV and BEV) throughout the life cycle are presented in Fig. 2.

For the GWP impacts of ICEV, the fuel cycle contributes to a large proportion (78.6%) considering the entire life cycle, which is mainly caused by gasoline burning in the PTW stage. The vehicle cycle accounts for the main contribution to the categories AP (62.3%), POCP (63.7%), and EP (78.2%), due to the materials related to the vehicle components, such as aluminum and steel. For the BEV, the contribution from the vehicle cycle and the fuel cycle is almost equal for the category GWP, since the electricity production takes place in the WTP stage and LIB manufacturing and replacement in the vehicle cycle. However, the vehicle cycle results in higher burden in the other categories (around 60% in AP, more 80% in POCP and EP) of the BEV, mostly due to the materials of vehicle components and LIBs, such as aluminum, lithium nickel cobalt manganese oxide, ethylene carbonate, printed circuit board, and steel.

In the total life cycle, the BEV has triple AP and five times more EP impacts than ICEV. Besides, BEV has 73.9% higher POCP impacts and 3.6% higher GWP impacts than the ICEV. For the vehicle cycle, the BEV shows higher impacts compared to the ICEV in four categories, 120~170% higher in GWP, AP, and POCP and more than 400% in EP, because of the material preparation, manufacturing, and replacement of the LIB. For the fuel cycle, the BEV has 21.5% lower GWP and 34.1% lower POCP impacts and 140~170% higher AP and EP impacts compared to the ICEV, which is due to the different types of fuel consumption. The other impact categories are also calculated according to the CML-IA method; detailed environmental impacts’ comparison results for the ICEV and BEV are presented in Table S15 in the Supplementary Information. In addition, the end-of-life (EoL) phase is modeled as part of the vehicle cycle. It is assumed that 100% of vehicles are collected, dismantled, and recycled. Thus, the production of primary materials, such as metals, plastics, rubber, and glass, are reduced by these amounts recovered during EoL. For further details, please see Section 8 in the Supplementary Information (EoL relative environmental impacts and life cycle environmental impacts including the EoL phase for the ICEV and BEV are shown in Table S18 and Table S19 in the Supplementary Information, respectively). The environmental results without considering LIB replacement are also provided in the supporting information (see chapter 4). They demonstrate the relevancy of considering the replacement, because without LIB replacement, the climate change impacts of BEV are lower compared to ICEV.

### Criticality Assessment

In this chapter, the results of the criticality assessment on global level applying the ESSENZ method (Section 3.2.1) and from Chinese perspective with an adapted ESSENZ approach (Section 3.2.2) are presented in detail.

#### Global Criticality Assessment by Applying ESSENZ

**Fig. 3:**
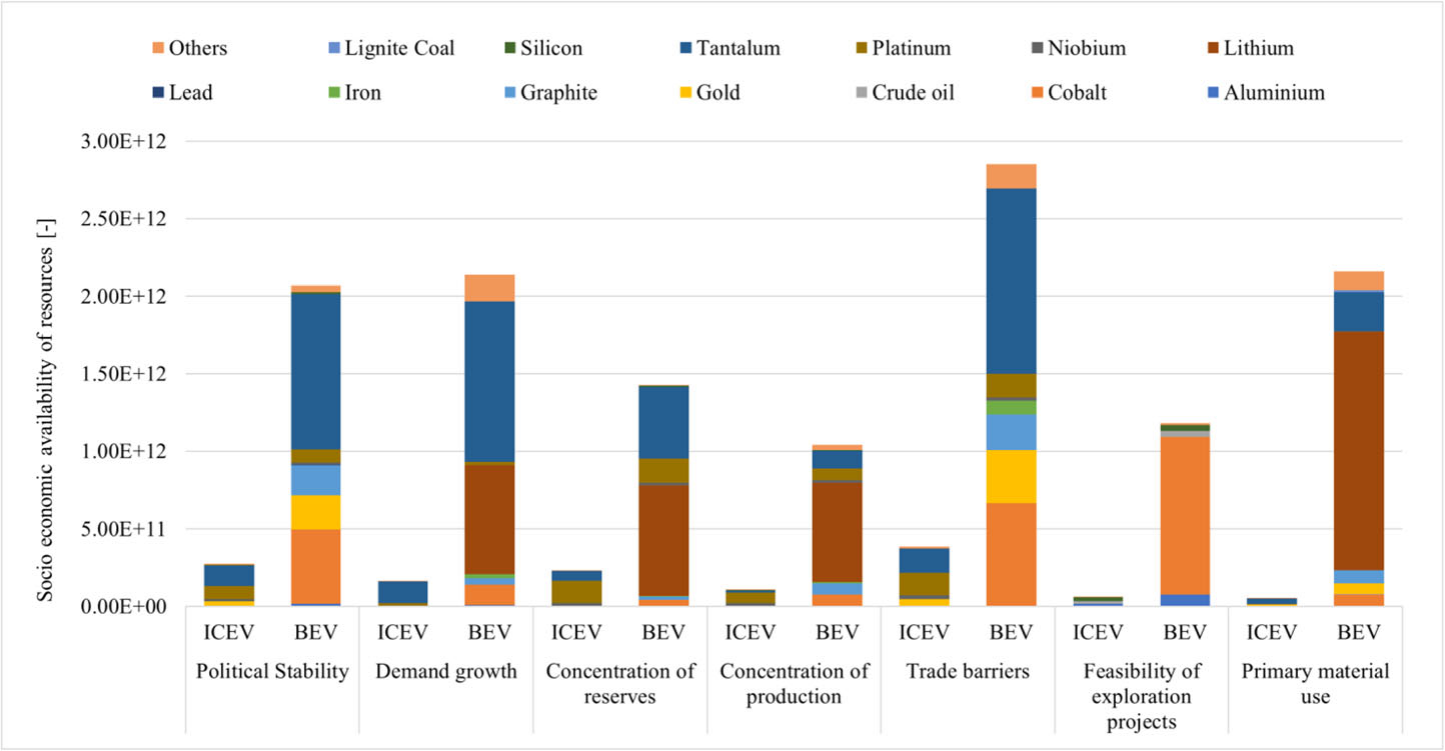
Comparison results of criticality assessment for the ICEV and BEV

Figure 3 shows the results of the criticality assessment (supply disruption) of the ICEV and BEV. The criticality is determined for the production of the vehicles as well as the use phase. Detailed comparison results of criticality aspects for the ICEV and BEV are presented in Table S16 in the Supplementary Information. With regard to the end-of-life phase, the criticality aspects for the ICEV and BEV are shown in Tables S20 and S21 in the Supplementary Information, respectively. The higher the numbers of the socio-economic availability (criticality) results are, the higher is the criticality of the resources used in the vehicles. Through the comparison between the ICEV and the BEV, it shows that the BEV has a much higher criticality than the ICEV in most of the categories: six times higher in the category concentration of reserves, seven times higher in the categories of trade barriers and company concentration, eight times higher in the categories mining capacity and political stability, nine times higher in the category price volatility, ten times higher in the categories of occurrence of co-production and concentration of production, more than ten times higher in the categories demand growth, twenty times higher in feasibility of exploration projects, and more than forty times higher in the category primary material use. Tantalum, lithium, cobalt, and gold, which are mainly used for electronics and LIBs, are the main contributors.

For the criticality in different life cycle stage, the results show that the vehicle cycle leads to higher potential supply disruptions than the fuel cycle in most categories for both the ICEV and the BEV. However, in the category price volatility for the ICEV, crude oil, which is used in the gasoline production phase in the fuel cycle, has the largest contribution (60.9%). While for the BEV, lignite coal, which is largely used in the electricity production phase in the fuel cycle, does not show the main contribution neither in price volatility (13.2%) nor in the other categories.

For both ICEV and BEV, tantalum contributes the largest proportion to the categories political stability (48.2% and 48.5%), demand growth (84.6% and 48.6%), and trade barriers (40.8% and 41.8%), because tantalum is mainly produced in Congo (Kinshasa) (41% of the global production) and Rwanda (28% of the global production); both countries face high political instability [67–69].

Due to the differences in the material composition of these two kinds of passenger vehicles, the main impacts in the other categories are contributed by different materials. For the ICEV, platinum has the main contribution (63.5%) to the concentration of reserves and production due to the fact that more than 80% of the world reserves are located in South Africa. For the BEV, tantalum and lithium dominate the categories concentration of reserves and production due to tantalum reserves and production dominated in Congo (Kinshasa) and Rwanda, leading to a high result for both categories [67–69]. Tantalum and lithium are the main contributors (together account for 81.3%) to the demand growth for the BEV. The demand for tantalum is driven by the growth of global electronics industry, which accounts for more than 50% of the total tantalum consumption [70]. In addition, global lithium consumption experienced rapid growth over the past few years with an 11% annual growth rate, due to the rapid development of the consumer electronics and electric vehicles [71–73].

As mentioned before, crude oil is the largest contributor of the price volatility for the ICEV, whereas for BEV, iron has the highest contribution (51.2%) due to changing world market prices. For the ICEV, niobium contributes most (63.3%) to the company concentration, because around 80% of all niobium is mined by only one company [74, 75]. For BEV, on the other hand, the category company concentration is dominated by graphite (84.5%), because 90% of the graphite worldwide is produced by companies in China, India, and Brazil.

Tantalum (41.8%), cobalt (23.3%), and gold (11.9%) are the top three contributors to the category trade barriers for the BEV. In Congo (Kinshasa) and Rwanda, the trade of tantalum is often highly influenced by rebel groups due to the extraction in artisanal mines, which have a non-transparent trade policy. Further, additional trade barriers are caused by the potential control of the artisanal production by the government [76].

In the category feasibility of exploration projects, impacts to BEV are overwhelming contributed by cobalt (86.2%), which is mainly located in Congo (Kinshasa) accounting for nearly 50% of the world reserves [77]. The high political unrest in Congo (Kinshasa) leads to the low feasibility of exploration projects [67, 68]. Lithium accounts for the 71.3% of the primary material use in the BEV. The small proportion of recycled lithium content is caused by the low recycling rate of waste lithium [50, 71]. The occurrence as a co-product of the BEV is mainly contributed by tantalum (28.0%) and cobalt (50.9%). The reason is that 33% of tantalum production occurs as the co-production of tantalum in polymetallic mines and tin slag [69], 50% of cobalt comes as the co-production of nickel, and 44% of cobalt produced as the by-products of copper and the other metals.

#### Adapted Approach Considering Chinese Production Patterns

In the following, the results for the categories trade barriers and political stability are shown considering Chinese production patterns. The results are compared to the global production results to determine significant differences.

**Fig. 4:**
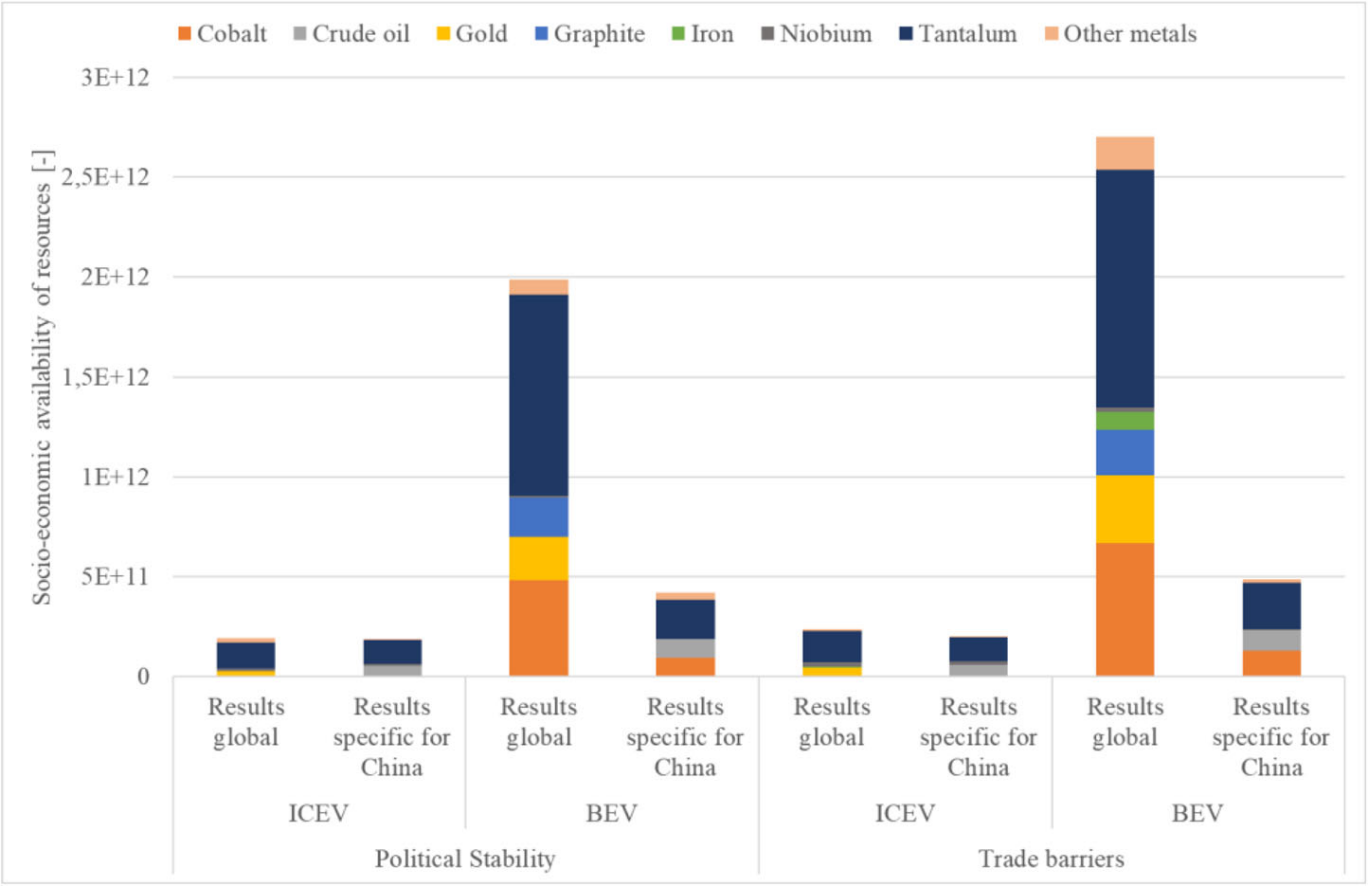
Comparison of global with Chinese-specific results for the BEV and ICEV

As it can be seen in Fig. 4 (and Table S17), the results specific for China are lower for both considered categories for the ICEV. Tantalum has a high potential supply disruption from a global as well as Chinese perspective. Tantalum is mainly mined outside of China and therefore has to be imported.

Potential supply disruptions of gold and platinum, which play in important role on a global level, are not relevant from a Chinse perspective. Almost 26% of the gold used in China is produced domestically and therefore considered risk free. Potential supply disruptions due to trade barriers and political instability of importing countries are rather low (e.g., imported from Australia).

The differences between the global results and Chinese-specific results for the potential supply disruptions for platinum are low, because South Africa and Russia are the main exporters. However, on a global level, also Zimbabwe, which has a high political instability, produces up to 10% of the world’s platinum. Since China is not importing from Zimbabwe, the global potential supply disruptions are higher.

Crude oil, which does not show significant potential supply disruptions on a global level (mainly due to many countries producing it), is potentially critical from a Chinese perspective. The domestically produced share of crude oil in China is only 5%. Most crude oil is imported from Saudi Arabia (25%) and Angola (19%), which show high trade barriers and political instability.

The results also show that the vehicle cycle leads to higher potential supply disruptions compared to the fuel cycle. However, the potential supply disruptions of crude oil have a higher influence in the fuel cycle of ICEV than BEV due to the higher consumption of fossil fuels in the use phase of ICEVs. Crude oil also plays a role for BEV due to its use in electricity production in China.

As seen in Fig. 4, the results specific for China are lower for both considered categories for the BEV. Tantalum and cobalt have high potential supply disruptions, because both are mainly imported from Congo, a country which is highly unstable and also imposes trade restrictions.

From a global point of view, again platinum and gold have high potential supply disruption compared to the Chinese-specific results, whereas the impact of crude oil is more relevant for China.

Since almost 100% of the graphite China uses is produced domestically, there is no risk associated. On a global level, however, the high production in China (by around 65%) leads to supply risks for other countries.

The results also show that the vehicle cycle of the BEV leads to much higher potential supply disruptions compared to the electricity (including emissions of upstream chains) used in the fuel cycle of the BEVs even though the current Chinese electricity mix (including crude oil, coal, and natural gas) is considered.

When comparing the results of ICEV and BEV for the Chinses perspective, it can be seen that the criticality of BEV is much higher than for ICEV, which follows the results on a global level. Again, tantalum and cobalt in the battery are the main contributors. Interestingly, even though the ICEV more strongly relies on crude oil in the fuel cycle, the associated criticality impacts are not more significant. The reason for that is that the current Chinese electricity mix was considered when determining criticality impacts of the fuel cycle.

### Synergy and Trade-Offs of Environmental Impacts and Criticality

The results of the environmental impacts show that the BEV has similar climate change impacts. This trend cannot be observed in the global criticality assessment, but in the Chinese-specific results. There have been discussions about the potential depletion of crude oil in the coming decade, which are another reason (besides climate change) alternative drivetrain technologies are investigated and high on the political agenda. However, these discussed shortages are predicted to arrive in 20 to 30 years and therefore are not reflected in the middle-term criticality assessment, which reflect potential supply disruptions for the next 5 years.

In the other environmental impacts, BEV performs worse than ICEV due vehicle components (mostly due to aluminum and steel) and the LIBs (due to, e.g., cobalt and lithium). For the criticality assessment, BEV also performs worse due to the used materials in the vehicle. However, the hotspot materials differ a bit: whereas aluminum and steel have been identified as environmental hotspots due to their overall used amount as well as high electricity use for processing for aluminum, they do not show a high criticality. However, aluminum should be watched for potential issues arising due to political instability and trade barrier. Tantalum has one of the highest criticality impacts, but is not an environmental hotspot. Synergies are seen for lithium and cobalt, which have high environmental impacts as well as a high criticality. Thus, it is advised to focus on these materials specifically with regard to reducing their criticality as well as environmental impacts. Overall, the biggest environmental and criticality impacts of ICEV are related to its use of fossil fuels only. However, as especially the global impacts due to climate change as well as predicted crude oil shortage are one of the most relevant issues of our time, the disadvantages of BEVs can hardly outweigh these aspects. However, it should be kept in mind that BEVs lead to higher environmental impacts (including climate change while considering LIB replacement) compared with ICEV and therefore should be further improved. When comparing the results of BEV with and without LIB replacement, it becomes clear that the replacement significantly impacts the environmental as well as criticality results, because without LIB replacement, the environmental impacts of BEV are lower compared to ICEV.

## Discussion

### Sensitivity Analysis

Lifetime service distance is the key factor for the life cycle environmental impacts. The sensitivity analysis of lifetime service distance is conducted by change of ± 10% of 150,000 km for both the ICEV and BEV. For the ICEV, the results show the ± 8% change in GWP, ± 4% change in AP and POCP, and ± 2% change in EP. For the BEV, the results show the ± 5% change in GWP, ± 4% change in AP, ± 2% change in POCP, and ± 1% change in EP.

According to the results in the Section 3, it is found that tantalum shows high potential supply disruption for both the ICEV and the BEV; crude oil has high criticality for the ICEV, especially in China; and cobalt has high potential supply disruptions for the BEV. Therefore, a single factor sensitivity analysis is performed to evaluate the impacts of tantalum, cobalt and crude oil on the criticality impacts for the ICEV and BEV. The sensitivity analysis of tantalum is conducted by changing the weights of ± 10% for the ICEV and BEV, respectively. The results show the ± 4% change in total criticality for both the ICEV and BEV. The sensitivity analysis of crude oil is conducted by changing the weights of ± 10% for the ICEV, which impacts the criticality results for the ICEV by ± 0.2%. The sensitivity analysis of cobalt is conducted by changing the weights of ± 10% for the BEV, which impacts the criticality results for the BEV by ± 1.4%. Therefore, the sensitivity analysis results show that lifetime service distance has a significant impact on life cycle GWP for both the ICEV and BEV. GWP benefits of the BEV become more pronounced with a longer lifetime distance. Besides, the amount of tantalum has a significant impact on the overall criticality impacts for both the ICEV and BEV.

### Uncertainty Analysis

Uncertainties in the vehicle material composition data, which are from the onsite surveys of 10 models in six Chinese leading automotive corporations in 2018, would lead to uncertainties in the presented results. However, it shows less than 3% difference of the material composition data in this study comparing with the literature data, such as Dai et al. [78].

Uncertainties of the ESSENZ CFs, which are discussed in the associated paper [39], are also leading to uncertainties in the presented results. The CFs specific for China for the two considered categories were determined based on data for import and domestic production in China. This data source could not be verified, and therefore a certain uncertainty might be associated with the used data.

Further, the applied criticality approach is a screening method only to identify a product’s or country’s dependency on certain raw materials [79, 80]. Thus, the results do not show the severity of an occurring supply disruption. The criticality assessment is also only done for raw materials but not intermediate products which might have been imported into China. As China is one of the main producing countries when it comes to production of electronic components as well as vehicle power batteries, this fact might not be as relevant as it would be for many other countries’ criticality profiles. The criticality hotspots are determined using data from the past years. Thus, future development cannot be predicted. The most recent example for this is the covid-19 pandemic, which leads to raw material shortcomings due to countries worldwide closing their borders and reducing overall production [81].

Further, pollution impacts like particulate matter (PM) were not part of the study, but are a relevant aspect, when comparing BEVs and ICEVs. Existing studies show that BEVs could reduce the life cycle and fuel cycle PM_2.5_ emissions compared with ICEVs with respect to Chinese energy-saving policies and actual emission-reduction techniques [14, 16, 82], while studies also find that the life cycle PM_2.5_ or PM_10_ emissions of BEVs are higher than those of ICEVs, mainly due to the high emission in the upstream industry process in electricity generation [19, 82]. Therefore, because of the inconsistence conclusions of the PM benefits in the existing studies, further studies are necessary to assess the PM pollution impacts.

## Conclusion

When analyzing the environmental impacts of BEV compared to ICEV considering Chinese inventory data, the results show that compared with the ICEV, the BEV has similar and partly higher environmental impacts, including GWP, AP, EP, and POCP, considering the whole life cycles. In the category GWP, the fuel cycle contributes to a large proportion for the ICEV while accounts for nearly 50% (close to the vehicle cycle) for the BEV. For both the ICEV and the BEV, the vehicle cycle accounts for the main contribution to the life cycle AP, POCP, and EP. When assessing the criticality aspects of BEV and ICEBs, it could be shown that BEVs have a higher criticality than the ICEV in most categories globally, especially in the primary material use, demand growth, and feasibility of exploration projects. Vehicle cycle leads to higher potential supply disruptions than the fuel cycle in most categories for both the ICEV and the BEV, with tantalum, lithium, and cobalt playing essential roles. The results specific for China are lower than the global results in both the trade barriers and political stability categories. In addition, gold and platinum, which play in important role on a global level, are not relevant from a Chinse perspective.

Thus, the results show that in the whole life cycle, the BEV performs similar and partly worse than the ICEV in all the environmental impact and criticality categories on a global as well as Chinese perspective. Thus, even though BEVs might be comparable with the ICEV in the climate change impacts, strategies have to be derived to deal with its possible criticality, especially challenges related to tantalum, lithium, and cobalt. It was demonstrated that the ICEV performs worse especially for crude oil use. However, as these aspects are that relevant, they outweigh the impacts of BEV in the other categories. Therefore, a shift from ICEB to BEV is still advised. However, this shift has to be carefully planned and timely to guarantee that criticality issues are managed. There are several strategies decision-makers might adapt, e.g., enter into trade agreements with countries mining critical materials or buying materials when they are available and stocking them up. However, as also environmental and social aspects are occurring during mining, future trade contracts should also include a transparent implementing of production standards, such as corporate social responsibility. As LIBs play an important role in the criticality of BEVs, improving the LIBs quality to reduce the replacement time during the use phase; establishing the monitoring, report, and verification (MRV) system to manage the LIBs quality; and developing the reuse and recycling management regulation for the disposal vehicles and LIBs base on the real condition are advised.

## Supplementary Information

ESM 1 (DOCX 138 kb)

## Data Availability

Data is provided in the supporting information.

## Nomenclature

AP: Acidification potential
BEVs: Battery electric vehicles
W_E_: Battery weight
CO_2_: Carbon dioxide
Mt CO_2_-eq: Carbon-dioxide equivalent
CFs: Characterization factors
CALCD: China automotive life cycle database
EVs: Electric vehicles
EoL: End-of-life
EP: Eutrophication potential
GWP: Global warming potential
HHI: Herfindahl-Hirschman Index
ESSENZ: Integrated method to assess resource efficiency
ICEVs: Internal combustion engine vehicles
LCA: Life cycle assessment
LiNi_x_Co_y_Mn_z_O_2_, NCM: Lithium nickel cobalt manganese oxide
LIBs: Lithium-ion power batteries
LIB: Lithium-ion power battery
POCP: Photochemical oxidant creation potential
PHEVs: Plug-in hybrid electric vehicles
PPI: Policy potential index
PTW: Pump to wheels
SWA: Sales weighted average
TWh: Terawatt-hours
E_T_: Test exhaust gas emission
F_T_: Test fuel consumption
W_V_: Vehicle weight
WTP: Well to pump
WGI: Worldwide governance indicators
