# Supplementary material for: Criticality Assessment of the Life Cycle of Passenger Vehicles Produced in China
Source: Circ Econ Sustain. 2021 Feb 23;1(1):435–55. doi: 10.1007/s43615-021-00012-5 (PMC7899740; doi:10.1007/s43615-021-00012-5)
Supplement: Supplementary file 1 — (DOCX 138 kb) [file 43615_2021_12_MOESM1_ESM.docx]

Supplementary Information

**Criticality assessment of the life cycle of passenger vehicles produced in China**

Xin Sun^123^, Vanessa Bach^4^*, Matthias Finkbeiner^4^, Jianxin Yang^23^

1 - China Automotive Technology and Research Center Co., Ltd, No. 68 East Xianfeng Road, Dongli District, Tianjin 300300, China

2 - State Key Laboratory of Urban and Regional Ecology, Research Center for Eco-Environmental Sciences, Chinese Academy of Sciences, No.18 Shuangqing road, Haidian District, Beijing 100085, China

3 - College of Resources and Environment, University of Chinese Academy of Sciences, No. 80 East Zhongguancun Road, Haidian District, Beijing 100190, China

4 - Technische Universität Berlin, Chair of Sustainable Enginnering, Straße des 17. Juni 135, 10623 Berlin

* corresponding author: Dr. Vanessa Bach, [vanessa.bach@tu-berlin.de](mailto:vanessa.bach@tu-berlin.de)

Content

[1 Paper outline 2](#_Toc63139936)

[2 ESSENZ method 2](#_Toc63139937)

[3 Life cycle inventory 3](#_Toc63139938)

[4 Environmental results without LIB replacement 4](#_Toc63139939)

[5 Data to derive category indicator results for considered resources 5](#_Toc63139940)

[6 Results of environmental impact categories according to the CML-IA method 10](#_Toc63139941)

[7 Results of criticality aspects for the ICEV and BEV 11](#_Toc63139942)

[8 Results of comparison of global with Chinese specific results for the BEV and ICEV 13](#_Toc63139943)

[9 End-of-Life phase results for the ICEV and BEV 14](#_Toc63139944)

[References 18](#_Toc63139945)

# Paper outline

In Figure S1 the paper outline is shown as well as associated goals.


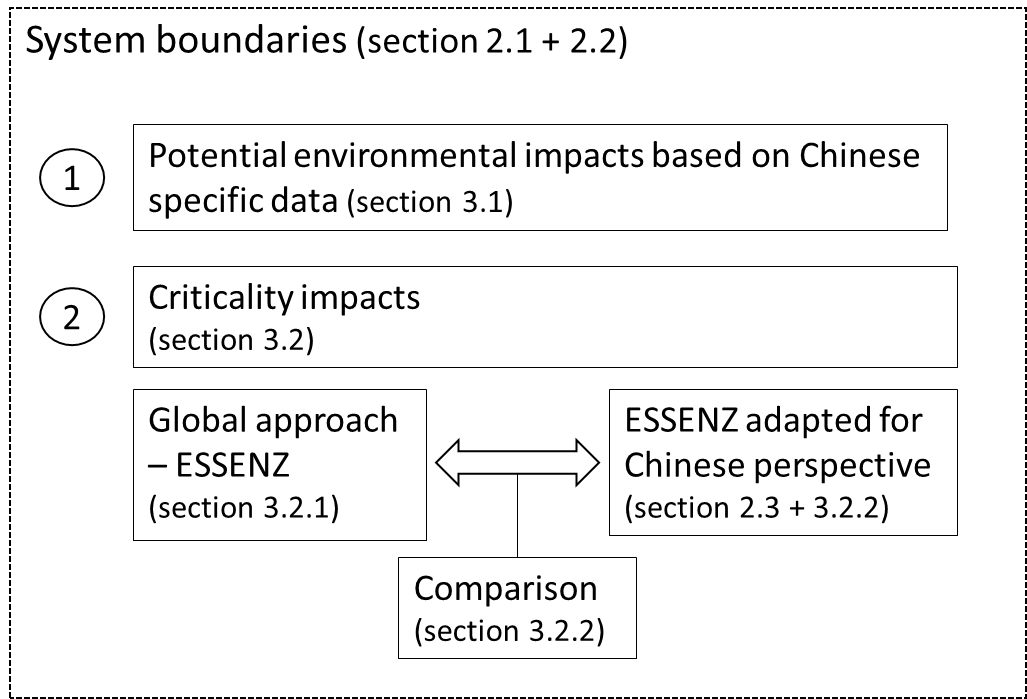


Figure S1: Overview of paper outline and goals

# ESSENZ method

The dimensions and categories of ESSENZ are shown in Table 1, including which were considered in this study.

Table S1: Overview on dimensions and categories of ESENZ

| **Sustainability dimension** | **Dimension of ESSENZ** | **Categories** | **Consideration in paper – global assessment** | **Consideration in paper – Chinese specific assessment** |
| --- | --- | --- | --- | --- |
| Economic | Criticality (potential supply disruption) | Concentration of production, reserves and company concentration | yes | no |
|  |  | Feasibility of exploration projects | yes | no |
|  |  | Political stability | yes | yes |
|  |  | Occurrence of co-product | yes | no |
|  |  | Mining capacity | yes | no |
|  |  | Primary material use | yes | no |
|  |  | Demand growth | yes | no |
|  |  | Price fluctuations | yes | no |
|  |  | Trade barriers | yes | yes |
|  | Physical availability | Abiotic resource depletion | yes | no |
| Environmental dimension | Environmental assessment | Climate change | yes | |
|  |  | Acidification | yes | |
|  |  | Eutrophication | yes | |
|  |  | Smog | yes | |
| Societal acceptance | Societal acceptance | Compliance with social standards | no | |
|  |  | Compliance with environmental standards | no | |

# Life cycle inventory

Table S1 Cumulative sales weighted average material composition for ICEVs and BEVs

| Material type | ICEV (kg) | BEV (kg) |
| --- | --- | --- |
| Steel | 774.59 | 740.26 |
| Cast Iron | 114.31 | 53.62 |
| Thermoplastics | 111.28 | 137.38 |
| Cast Aluminum | 86.28 | 182.60 |
| Rubber | 65.58 | 86.30 |
| Ceramics / glass | 39.31 | 50.30 |
| Duromers | 22.73 | 32.64 |
| Wrought Aluminum | 21.60 | 64.70 |
| Coolant | 15.83 | 11.62 |
| Copper and copper alloys | 14.47 | 60.07 |
| Textiles | 14.08 | 13.71 |
| Lead | 13.88 | 8.83 |
| Lithium nickel cobalt manganite | 0.00 | 77.06 |
| Graphite/Carbon | 0.00 | 40.95 |
| Modified organic natural materials | 2.19 | 34.01 |
| Others | 45.94 | 64.66 |

# Environmental results without LIB replacement

In the following the results without the LIB replacement are shown (see Table S2).

Table S2: Life cycle environmental impacts comparison for the ICEV and BEV without LIB replacement

| Impact category | Unit | ICEV | BEV |
| --- | --- | --- | --- |
| Abiotic depletion | kg Sb e | 0.4 | 0.8 |
| Abiotic depletion (fossil fuels) | GJ | 603.9 | 284.7 |
| Global warming (GWP100a) | t CO_2_ e | 34.4 | 30.1 |
| Ozone layer depletion (ODP) | kg CFC-11 e | 0.1 | 0.1 |
| Human toxicity | t 1.4-DB e | 3.6 | 9.1 |
| Fresh water aquatic ecotox. | t 1.4-DB e | 4.6 | 19.0 |
| Marine aquatic ecotoxicity | t 1.4-DB e | 40744.3 | 88730.8 |
| Terrestrial ecotoxicity | kg 1.4-DB e | 14.0 | 51.9 |
| Photochemical oxidation | kg C_2_H_4_ e | 6.2 | 8.5 |
| Acidification | kg SO_2_ e | 44.8 | 95.0 |
| Eutrophication | kg PO_4_^3-^ e | 6.6 | 21.5 |

For the GWP impacts of ICEV, the fuel cycle contributes to a large proportion (78.6%) considering the entire life cycle, which is mainly caused by gasoline burning in the PTW stage. The vehicle cycle accounts for the main contribution to the categories AP (62.3%), POCP (63.7%) and EP (78.2%), due to the materials related to the vehicle components, such as aluminum and steel. For the BEV, the fuel cycle is the main contributor (59.1%) for the category GWP, since the electricity production takes place in the WTP stage. The vehicle cycle results in higher burden (around 80%) in the categories POCP and EP, mostly due to the materials of vehicle components and LIBs, such as aluminum, lithium nickel cobalt manganese oxide, ethylene carbonate, printed circuit board and steel.

In the total life cycle, the BEV has twice more AP and three times more EP impacts than ICEV. Besides, BEV has 36.9% higher POCP impacts but 12.5% lower GWP impacts than the ICEV. For the vehicle cycle, the BEV shows higher impacts compared to the ICEV in four categories, around 70% in GWP, AP and POCP and more than 200% in EP, because of the material preparation and manufacturing of the LIB. For the fuel cycle, the BEV has 20%~35% lower GWP and POCP impacts and more than 140% higher AP and EP impacts compared to the ICEV, which is due to the different types of fuel consumption.

# Data to derive category indicator results for considered resources

Table S 2: Overview of imports, share of imports category indicator results for political stability and trade barriers for crude oil

| Country | Imports 2015 [Mt] | Share of imports  (in %) | Category indicator for political stability | Category indicator for trade barriers |
| --- | --- | --- | --- | --- |
| Other countries | 67.26 | 0.03 |  |  |
| Iran | 21411.55 | 9.97 | 30.15 | 44.3 |
| Iraq | 23512.94 | 10.95 | 39.70 | 44.8 |
| Oman | 25469.56 | 11.86 | 21.92 | 34.8 |
| Russia | 24348.42 | 11.34 | 32.06 | 43.1 |
| Saudi Arabia | 53902.85 | 25.10 | 58.88 | 82.0 |
| United Arab Emirates | 10275.85 | 4.78 | 6.08 | 11.4 |
| Angola | 40009.85 | 18.63 | 58.61 | 89.4 |
| Venezuela | 15551.97 | 7.24 | 25.88 | 34.4 |
| China | 211.00 | 0.10 |  |  |
|  |  |  | 273.28 | 384.18 |

Table S 3: Overview of imports, share of imports category indicator results for political stability and trade barriers for lignite

| Country | Imports 2015 [Mt] | Share of imports  (in %) | Category indicator for political stability | Category indicator for trade barriers |
| --- | --- | --- | --- | --- |
| Other countries | 0.41 | 0.21 |  |  |
| Indonesia | 57.95 | 28.93 | 67.93 | 95.5 |
| Philippines | 1.93 | 0.96 | 2.20 | 3.3 |
| China | 140.00 | 69.90 |  |  |
|  |  |  | 70.13 | 98.83 |

Table S 4: Overview of imports, share of imports category indicator results for political stability and trade barriers for hard coal

| Country | Imports 2015 [Mt] | Share of imports  (in %) | Category indicator for political stability | Category indicator for trade barriers |
| --- | --- | --- | --- | --- |
| Other countries | 1.75 | 0.05 |  |  |
| Vietnam | 13.12 | 0.37 | 0.92 | 1.2 |
| Australia | 55.13 | 1.56 | 0.53 | 3.9 |
| Russia | 10.95 | 0.31 | 0.87 | 1.2 |
| Indonesia | 38.91 | 1.10 | 2.58 | 3.6 |
| Mongolia | 1.41 | 0.04 | 0.09 | 0.2 |
| North Korea | 16.48 | 0.47 | 1.80 | 1.7 |
| South Africa | 12.74 | 0.36 | 0.66 | 1.1 |
| US | 2.23 | 0.06 | 0.04 | 0.1 |
| China | 3387.20 | 95.69 |  |  |
|  |  |  | 7.49 | 13.02 |

Table S 5: Overview of imports, share of imports category indicator results for political stability and trade barriers for aluminum

| Country | Imports 2015 [kt] | Share of imports  (in %) | Category indicator for political stability | Category indicator for trade barriers |
| --- | --- | --- | --- | --- |
| Other countries | 3216.07 | 10.18 |  |  |
| India | 5.32 | 0.02 | 0.04 | 0.1 |
| Indonesia | 47.39 | 0.15 | 0.35 | 0.5 |
| Australia | 14.29 | 0.05 | 0.02 | 0.1 |
| China | 28316.7 | 89.61 |  |  |
|  |  |  | 0.41 | 0.67 |

Table S 6: Overview of imports, share of imports category indicator results for political stability and trade barriers for chromium

| Country | Imports 2015 [kt] | Share of imports  (in %) | Category indicator for political stability | Category indicator for trade barriers |
| --- | --- | --- | --- | --- |
| Other countries | 1113.55 | 9.06 |  |  |
| Australia | 408.07 | 3.32 | 1.12 | 8.3 |
| Oman | 685.55 | 5.58 | 10.31 | 16.4 |
| Pakistan | 484.93 | 3.94 | 12.39 | 16.1 |
| South Africa | 6738.23 | 54.82 | 100.97 | 168.7 |
| Turkey | 1985.56 | 16.15 | 36.48 | 49.8 |
| Albania | 676.50 | 5.50 | 11.23 | 17.0 |
| China | 200.0 | 1.63 |  |  |
|  |  |  | 172.50 | 276.28 |

Table S 7: Overview of imports, share of imports category indicator results for political stability and trade barriers for copper

| Country | Imports 2015 [kt] | Share of imports  (in %) | Category indicator for political stability | Category indicator for trade barriers |
| --- | --- | --- | --- | --- |
| Other countries | 2735.22 | 15.44 |  |  |
| Mongolia | 566.43 | 3.2 | 6.90 | 12.6 |
| Australia | 891.51 | 5.03 | 1.70 | 12.6 |
| Chile | 2839.93 | 16.03 | 13.93 | 37.4 |
| Peru | 1920.26 | 10.84 | 24.47 | 33.1 |
| USA | 557.19 | 3.14 | 2.11 | 7.4 |
| Mexico | 558.45 | 3.15 | 7.31 | 9.6 |
| China | 7649.10 | 43.17 |  |  |
|  |  |  | 56.42 | 112.82 |

Table S 8: Overview of imports, share of imports category indicator results for political stability and trade barriers for iron

| Country | Imports 2015 [mt] | Share of imports  (in %) | Category indicator for political stability | Category indicator for trade barriers |
| --- | --- | --- | --- | --- |
| Other countries | 152.66 | 6.54 |  |  |
| Australia | 416.81 | 17.86 | 6.03 | 44.7 |
| Iran | 22.42 | 0.96 | 2.91 | 4.3 |
| India | 11.64 | 0.50 | 1.13 | 1.8 |
| Indonesia | 17.50 | 0.75 | 1.76 | 2.5 |
| South Africa | 43.02 | 1.84 | 3.40 | 5.7 |
| Brazil | 155.05 | 6.64 | 14.72 | 25.3 |
| China | 1.514.24 | 64.90 |  |  |
|  |  |  | 29.94 | 84.27 |

Table S 9: Overview of imports, share of imports category indicator results for political stability and trade barriers for lead

| Country | Imports 2015 [kt] | Share of imports  (in %) | Category indicator for political stability | Category indicator for trade barriers |
| --- | --- | --- | --- | --- |
| Other countries | 347.47 | 3.91 |  |  |
| Iraq | 37.89 | 0.43 | 1.54 | 1.7 |
| KOREA. DEM. REP. | 117.55 | 1.32 | 5.12 | 4.7 |
| South Africa | 54.00 | 0.61 | 1.12 | 1.9 |
| Turkey | 94.29 | 1.06 | 2.39 | 3.3 |
| Australia | 144.64 | 1.63 | 0.55 | 4.1 |
| Peru | 104.73 | 1.18 | 2.66 | 3.6 |
| USA | 216.19 | 2.43 | 1.63 | 5.7 |
| Mexico | 62.20 | 0.70 | 1.62 | 2.1 |
| Germany | 44.36 | 0.50 | 0.19 | 1.1 |
| Poland | 54.21 | 0.61 | 0.68 | 1.6 |
| Russia | 215.30 | 2.42 | 6.84 | 9.2 |
| China | 7404.00 | 83.22 |  |  |
|  |  |  | 24.33 | 38.99 |

Table S 10: Overview of imports, share of imports category indicator results for political stability and trade barriers for magnesium

| Country | Imports 2015 [kt] | Share of imports  (in %) | Category indicator for political stability | Category indicator for trade barriers |
| --- | --- | --- | --- | --- |
| Other countries | 7.83 | 0.77 |  |  |
| Australia | 0.19 | 0.02 | 0.01 | 0.0 |
| Japan | 5.80 | 0.57 | 0.32 | 1.3 |
| North Korea | 130.81 | 12.84 | 49.77 | 45.8 |
| Netherlands | 0.18 | 0.02 | 0.00 | 0.0 |
| China | 873.60 | 85.78 |  |  |
|  |  |  | 50.10 | 47.20 |

Table S 11: Overview of imports. share of imports category indicator results for political stability and trade barriers for natural gas

| Country | Imports 2015 [kt] | Share of imports  (in %) | Category indicator for political stability | Category indicator for trade barriers |
| --- | --- | --- | --- | --- |
| Other countries | 267.15 | 0.26 |  |  |
| Turkmenistan | 17709.77 | 17.06 | 59.10 | 44.2 |
| Uzbekistan | 2097.34 | 2.02 | 6.63 | 6.9 |
| China | 83743.48 | 80.66 |  |  |
|  |  |  | 65.73 | 51.11 |

Table S 12: Overview of imports, share of imports category indicator results for political stability and trade barriers for nickel

| Country | Imports 2015 [kt] | Share of imports  (in %) | Category indicator for political stability | Category indicator for trade barriers |
| --- | --- | --- | --- | --- |
| Other countries | 493.95 | 0.69 |  |  |
| Indonesia | 41051.55 | 57.43 | 134.83 | 189.6 |
| Philippines | 29694.29 | 41.54 | 94.91 | 143.9 |
| China | 246.70 | 0.35 |  |  |
|  |  |  | 229.74 | 333.50 |

Table S 13: Overview of imports, share of imports category indicator results for political stability and trade barriers for titanium

| Country | Imports 2015 [kt] | Share of imports  (in %) | Category indicator for political stability | Category indicator for trade barriers |
| --- | --- | --- | --- | --- |
| Other countries | 237.88 | 7.63 |  |  |
| India | 435.62 | 13.98 | 31.57 | 51.6 |
| Korea | 152.80 | 4.90 | 6.06 | 12.6 |
| Vietnam | 587.07 | 18.84 | 46.62 | 63.0 |
| Mozambique | 134.67 | 4.32 | 11.71 | 16.1 |
| Australia | 583.99 | 18.74 | 6.32 | 46.9 |
| Russia | 133.67 | 4.30 | 12.13 | 16.3 |
| China | 850.00 | 27.28 |  |  |
|  |  |  | 114.41 | 206.54 |

Table S 14: Overview of imports, share of imports category indicator results for political stability and trade barriers for zinc

| Country | Imports 2015 [kt] | Share of imports  (in %) | Category indicator for political stability | Category indicator for trade barriers |
| --- | --- | --- | --- | --- |
| Other Countries | 220.92 | 2.92 |  |  |
| Mongolia | 116.18 | 1.53 | 3.31 | 6.0 |
| Turkey | 110.49 | 1.46 | 3.29 | 3.1 |
| Australia | 808.02 | 10.67 | 3.60 | 26.7 |
| Peru | 366.85 | 4.84 | 10.93 | 3.1 |
| Canada | 366.85 | 4.84 | 1.20 | 11.9 |
| China | 5807.00 | 76.66 |  |  |
|  |  |  | 22.34 | 50.75 |

# Results of environmental impact categories according to the CML-IA method

Table S 15 Life cycle environmental impacts comparison for the ICEV and BEV

| Impact category | Unit | ICEV | BEV |
| --- | --- | --- | --- |
| Abiotic depletion | kg Sb e | 0.4 | 0.9 |
| Abiotic depletion (fossil fuels) | GJ | 603.9 | 339.8 |
| Global warming (GWP100a) | t CO_2_ e | 34.4 | 35.6 |
| Ozone layer depletion (ODP) | kg CFC-11 e | 0.1 | 0.1 |
| Human toxicity | t 1.4-DB e | 3.6 | 12.2 |
| Fresh water aquatic ecotox. | t 1.4-DB e | 4.6 | 27.9 |
| Marine aquatic ecotoxicity | t 1.4-DB e | 40744.3 | 124548.3 |
| Terrestrial ecotoxicity | kg 1.4-DB e | 14.0 | 80.8 |
| Photochemical oxidation | kg C_2_H_4_ e | 6.2 | 10.8 |
| Acidification | kg SO_2_ e | 44.8 | 119.9 |
| Eutrophication | kg PO_4_^3-^ e | 6.6 | 32.3 |

# Results of criticality aspects for the ICEV and BEV

Table S 16: Criticality aspects comparison for the ICEV and BEV

|  | Political Stability | | Demand growth | | Mining capacity | | Concentration of reserves | | Concentration of production | | Trade barriers | | Feasibility of exploration projects | | Price volatility | | occurrence of co-production | | Primary material use | | Company concentration | |
| --- | --- | --- | --- | --- | --- | --- | --- | --- | --- | --- | --- | --- | --- | --- | --- | --- | --- | --- | --- | --- | --- | --- |
|  | ICEV | BEV | ICEV | BEV | ICEV | BEV | ICEV | BEV | ICEV | BEV | ICEV | BEV | ICEV | BEV | ICEV | BEV | ICEV | BEV | ICEV | BEV | ICEV | BEV |
| Aluminium | 2.8E+09 | 1.5E+10 | 1.2E+09 | 6.4E+09 | 0.0E+00 | 0.0E+00 | 1.6E+08 | 8.6E+08 | 7.8E+08 | 4.1E+09 | 0.0E+00 | 0.0E+00 | 1.4E+10 | 7.6E+10 | 0.0E+00 | 0.0E+00 | 0.0E+00 | 0.0E+00 | 0.0E+00 | 0.0E+00 | 0.0E+00 | 0.0E+00 |
| Cadmium | 1.0E+07 | 8.5E+06 | 0.0E+00 | 0.0E+00 | 2.7E+04 | 2.3E+04 | 0.0E+00 | 0.0E+00 | 3.6E+05 | 3.0E+05 | 0.0E+00 | 0.0E+00 | 0.0E+00 | 0.0E+00 | 1.8E+06 | 1.5E+06 | 2.4E+06 | 2.0E+06 | 2.8E+06 | 2.4E+06 | 0.0E+00 | 0.0E+00 |
| Calcium | 3.5E+06 | 1.6E+08 | 0.0E+00 | 0.0E+00 | 0.0E+00 | 0.0E+00 | 0.0E+00 | 0.0E+00 | 1.5E+06 | 6.7E+07 | 4.5E+06 | 2.0E+08 | 0.0E+00 | 0.0E+00 | 0.0E+00 | 0.0E+00 | 0.0E+00 | 0.0E+00 | 2.1E+06 | 9.1E+07 | 3.3E+05 | 1.5E+07 |
| Chromium | 6.3E+05 | 6.9E+06 | 1.4E+05 | 1.6E+06 | 1.8E+04 | 2.0E+05 | 1.9E+05 | 2.1E+06 | 1.0E+05 | 1.1E+06 | 9.9E+05 | 1.1E+07 | 2.5E+06 | 2.7E+07 | 0.0E+00 | 0.0E+00 | 0.0E+00 | 0.0E+00 | 2.5E+05 | 2.7E+06 | 0.0E+00 | 0.0E+00 |
| Cobalt | 0.0E+00 | 4.8E+11 | 0.0E+00 | 1.3E+11 | 0.0E+00 | 7.2E+08 | 0.0E+00 | 4.3E+10 | 0.0E+00 | 7.2E+10 | 0.0E+00 | 6.7E+11 | 0.0E+00 | 1.0E+12 | 0.0E+00 | 0.0E+00 | 0.0E+00 | 5.1E+10 | 0.0E+00 | 7.7E+10 | 0.0E+00 | 0.0E+00 |
| Copper | 0.0E+00 | 0.0E+00 | 0.0E+00 | 0.0E+00 | 9.4E+06 | 2.4E+08 | 1.3E+08 | 3.3E+09 | 0.0E+00 | 0.0E+00 | 0.0E+00 | 0.0E+00 | 0.0E+00 | 0.0E+00 | 0.0E+00 | 0.0E+00 | 1.0E+08 | 2.6E+09 | 6.9E+08 | 1.8E+10 | 0.0E+00 | 0.0E+00 |
| Crude oil | 0.0E+00 | 0.0E+00 | 0.0E+00 | 0.0E+00 | 9.1E+06 | 1.5E+07 | 0.0E+00 | 0.0E+00 | 0.0E+00 | 0.0E+00 | 0.0E+00 | 0.0E+00 | 2.1E+10 | 3.5E+10 | 6.4E+09 | 1.1E+10 | 0.0E+00 | 0.0E+00 | 2.3E+09 | 4.0E+09 | 7.2E+07 | 1.2E+08 |
| Gold | 2.9E+10 | 2.2E+11 | 0.0E+00 | 0.0E+00 | 5.0E+08 | 3.8E+09 | 0.0E+00 | 0.0E+00 | 0.0E+00 | 0.0E+00 | 4.5E+10 | 3.4E+11 | 0.0E+00 | 0.0E+00 | 0.0E+00 | 0.0E+00 | 0.0E+00 | 0.0E+00 | 8.7E+09 | 6.6E+10 | 0.0E+00 | 0.0E+00 |
| Graphite | 0.0E+00 | 1.9E+11 | 0.0E+00 | 3.9E+10 | 0.0E+00 | 0.0E+00 | 0.0E+00 | 2.0E+10 | 0.0E+00 | 7.0E+10 | 0.0E+00 | 2.3E+11 | 0.0E+00 | 0.0E+00 | 0.0E+00 | 0.0E+00 | 0.0E+00 | 0.0E+00 | 0.0E+00 | 8.5E+10 | 0.0E+00 | 4.3E+10 |
| Iron | 0.0E+00 | 0.0E+00 | 8.1E+08 | 2.9E+10 | 1.6E+07 | 5.9E+08 | 8.5E+07 | 3.1E+09 | 2.5E+08 | 9.0E+09 | 2.5E+09 | 9.1E+10 | 0.0E+00 | 0.0E+00 | 1.3E+09 | 4.8E+10 | 0.0E+00 | 0.0E+00 | 0.0E+00 | 0.0E+00 | 0.0E+00 | 0.0E+00 |
| Lead | 5.1E+09 | 4.1E+09 | 0.0E+00 | 0.0E+00 | 1.1E+08 | 8.9E+07 | 5.7E+08 | 4.6E+08 | 9.3E+08 | 7.5E+08 | 0.0E+00 | 0.0E+00 | 0.0E+00 | 0.0E+00 | 1.9E+09 | 1.5E+09 | 2.7E+08 | 2.2E+08 | 0.0E+00 | 0.0E+00 | 0.0E+00 | 0.0E+00 |
| Lignite Coal | 0.0E+00 | 0.0E+00 | 0.0E+00 | 0.0E+00 | 0.0E+00 | 0.0E+00 | 2.8E+07 | 1.5E+09 | 0.0E+00 | 0.0E+00 | 0.0E+00 | 0.0E+00 | 0.0E+00 | 0.0E+00 | 2.3E+08 | 1.2E+10 | 0.0E+00 | 0.0E+00 | 3.0E+08 | 1.6E+10 | 9.2E+06 | 4.8E+08 |
| Lithium | 0.0E+00 | 0.0E+00 | 0.0E+00 | 7.0E+11 | 0.0E+00 | 0.0E+00 | 0.0E+00 | 7.1E+11 | 0.0E+00 | 6.4E+11 | 0.0E+00 | 0.0E+00 | 0.0E+00 | 0.0E+00 | 0.0E+00 | 0.0E+00 | 0.0E+00 | 0.0E+00 | 0.0E+00 | 1.5E+12 | 0.0E+00 | 0.0E+00 |
| Magnesium | 4.8E+07 | 2.0E+08 | 1.3E+08 | 5.5E+08 | 0.0E+00 | 0.0E+00 | 2.3E+06 | 9.7E+06 | 2.6E+07 | 1.1E+08 | 5.4E+07 | 2.3E+08 | 0.0E+00 | 0.0E+00 | 0.0E+00 | 0.0E+00 | 1.6E+06 | 6.9E+06 | 0.0E+00 | 0.0E+00 | 9.4E+05 | 3.9E+06 |
| Manganese | 0.0E+00 | 0.0E+00 | 1.7E+08 | 6.9E+08 | 2.7E+06 | 1.1E+07 | 4.8E+07 | 1.9E+08 | 5.7E+07 | 2.3E+08 | 1.1E+09 | 4.4E+09 | 2.6E+09 | 1.1E+10 | 0.0E+00 | 0.0E+00 | 0.0E+00 | 0.0E+00 | 0.0E+00 | 0.0E+00 | 0.0E+00 | 0.0E+00 |
| Natural Gas | 0.0E+00 | 0.0E+00 | 0.0E+00 | 0.0E+00 | 0.0E+00 | 0.0E+00 | 0.0E+00 | 0.0E+00 | 0.0E+00 | 0.0E+00 | 0.0E+00 | 0.0E+00 | 1.7E+08 | 3.2E+09 | 6.7E+07 | 1.3E+09 | 0.0E+00 | 0.0E+00 | 2.9E+07 | 5.5E+08 | 9.0E+05 | 1.7E+07 |
| Nickel | 0.0E+00 | 0.0E+00 | 2.8E+08 | 5.3E+10 | 1.3E+06 | 2.4E+08 | 0.0E+00 | 0.0E+00 | 0.0E+00 | 0.0E+00 | 4.9E+08 | 9.4E+10 | 0.0E+00 | 0.0E+00 | 9.2E+07 | 1.8E+10 | 1.4E+07 | 2.6E+09 | 0.0E+00 | 0.0E+00 | 0.0E+00 | 0.0E+00 |
| Niobium | 1.1E+10 | 1.1E+10 | 0.0E+00 | 0.0E+00 | 0.0E+00 | 0.0E+00 | 1.9E+10 | 1.9E+10 | 1.8E+10 | 1.7E+10 | 2.3E+10 | 2.3E+10 | 0.0E+00 | 0.0E+00 | 0.0E+00 | 0.0E+00 | 5.1E+08 | 5.0E+08 | 4.2E+09 | 4.1E+09 | 4.3E+09 | 4.3E+09 |
| Phosphorus | 1.5E+06 | 1.3E+06 | 3.2E+05 | 2.7E+05 | 0.0E+00 | 0.0E+00 | 7.0E+05 | 5.9E+05 | 2.9E+05 | 2.4E+05 | 1.9E+06 | 1.6E+06 | 1.2E+07 | 1.0E+07 | 5.1E+05 | 4.3E+05 | 0.0E+00 | 0.0E+00 | 7.2E+05 | 6.0E+05 | 1.3E+05 | 1.1E+05 |
| Platinum | 8.2E+10 | 8.6E+10 | 2.0E+10 | 2.1E+10 | 0.0E+00 | 0.0E+00 | 1.5E+11 | 1.5E+11 | 6.8E+10 | 7.2E+10 | 1.5E+11 | 1.5E+11 | 0.0E+00 | 0.0E+00 | 0.0E+00 | 0.0E+00 | 4.2E+09 | 4.4E+09 | 0.0E+00 | 0.0E+00 | 1.8E+09 | 1.9E+09 |
| Sand and Gravel | 0.0E+006.68E+09 | 0.0E+009.72E+09 | 2.2E+090.00E+00 | 1.1E+110.00E+00 | 0.0E+000.00E+00 | 0.0E+000.00E+00 | 0.0E+002.61E+09 | 0.0E+003.80E+09 | 6.0E+083.00E+09 | 3.2E+104.36E+09 | 0.0E+000.00E+00 | 0.0E+000.00E+00 | 0.0E+002.23E+10 | 0.0E+003.25E+10 | 0.0E+000.00E+00 | 0.0E+000.00E+00 | 9.9E+070.00E+00 | 5.2E+090.00E+00 | 1.6E+090.00E+00 | 8.5E+100.00E+00 | 0.0E+006.81E+08 | 0.0E+009.91E+08 |
| Silicon | 6.7E+093.67E+09 | 1.2E+102.67E+10 | 0.0E+000.00E+00 | 0.0E+000.00E+00 | 0.0E+005.17E+07 | 0.0E+003.76E+08 | 2.6E+090.00E+00 | 4.5E+090.00E+00 | 3.0E+090.00E+00 | 5.2E+090.00E+00 | 0.0E+005.51E+09 | 0.0E+004.01E+10 | 2.2E+100.00E+00 | 3.8E+100.00E+00 | 0.0E+000.00E+00 | 0.0E+000.00E+00 | 0.0E+007.07E+08 | 0.0E+005.15E+09 | 0.0E+001.23E+09 | 0.0E+008.98E+09 | 6.8E+080.00E+00 | 1.2E+090.00E+00 |
| Silver | 3.7E+090.00E+00 | 2.8E+100.00E+00 | 0.0E+000.00E+00 | 0.0E+000.00E+00 | 5.2E+070.00E+00 | 3.9E+080.00E+00 | 0.0E+000.00E+00 | 0.0E+000.00E+00 | 0.0E+000.00E+00 | 0.0E+000.00E+00 | 5.5E+090.00E+00 | 4.2E+103.71E+08 | 0.0E+000.00E+00 | 0.0E+000.00E+00 | 0.0E+000.00E+00 | 0.0E+000.00E+00 | 7.1E+080.00E+00 | 5.3E+091.29E+07 | 1.2E+090.00E+00 | 9.3E+091.73E+08 | 0.0E+000.00E+00 | 0.0E+000.00E+00 |
| Sodium | 0.0E+001.33E+11 | 0.0E+009.68E+11 | 0.0E+001.37E+11 | 0.0E+001.00E+12 | 0.0E+000.00E+00 | 0.0E+000.00E+00 | 0.0E+006.13E+10 | 0.0E+004.47E+11 | 0.0E+001.54E+10 | 0.0E+001.12E+11 | 0.0E+001.58E+11 | 7.4E+081.15E+12 | 0.0E+000.00E+00 | 0.0E+000.00E+00 | 0.0E+000.00E+00 | 0.0E+000.00E+00 | 0.0E+003.72E+09 | 2.6E+072.71E+10 | 0.0E+003.30E+10 | 3.5E+082.40E+11 | 0.0E+000.00E+00 | 0.0E+000.00E+00 |
| Tantalum | 1.3E+111.99E+09 | 1.0E+127.67E+09 | 1.4E+110.00E+00 | 1.0E+120.00E+00 | 0.0E+004.23E+07 | 0.0E+001.63E+08 | 6.1E+100.00E+00 | 4.6E+110.00E+00 | 1.5E+101.64E+08 | 1.2E+116.32E+08 | 1.6E+112.42E+09 | 1.2E+129.32E+09 | 0.0E+000.00E+00 | 0.0E+000.00E+00 | 0.0E+000.00E+00 | 0.0E+000.00E+00 | 3.7E+090.00E+00 | 2.8E+100.00E+00 | 3.3E+105.01E+08 | 2.5E+111.93E+09 | 0.0E+000.00E+00 | 0.0E+000.00E+00 |
| Tinn | 2.0E+090.00E+00 | 8.7E+090.00E+00 | 0.0E+000.00E+00 | 0.0E+000.00E+00 | 4.2E+070.00E+00 | 1.8E+080.00E+00 | 0.0E+009.47E+06 | 0.0E+001.06E+07 | 1.6E+080.00E+00 | 7.1E+080.00E+00 | 2.4E+092.89E+08 | 1.1E+103.22E+08 | 0.0E+000.00E+00 | 0.0E+000.00E+00 | 0.0E+001.87E+08 | 0.0E+002.08E+08 | 0.0E+000.00E+00 | 0.0E+000.00E+00 | 5.0E+080.00E+00 | 2.2E+090.00E+00 | 0.0E+000.00E+00 | 0.0E+000.00E+00 |
| Titanium | 0.0E+001.05E+09 | 0.0E+003.19E+09 | 0.0E+000.00E+00 | 0.0E+000.00E+00 | 0.0E+002.52E+07 | 0.0E+007.69E+07 | 9.7E+066.49E+07 | 1.2E+071.98E+08 | 0.0E+008.04E+07 | 0.0E+002.45E+08 | 3.0E+081.69E+09 | 3.7E+085.15E+09 | 0.0E+000.00E+00 | 0.0E+000.00E+00 | 1.9E+083.63E+08 | 2.4E+081.11E+09 | 0.0E+005.42E+07 | 0.0E+001.65E+08 | 0.0E+004.39E+08 | 0.0E+001.34E+09 | 0.0E+000.00E+00 | 0.0E+000.00E+00 |
| Zinc | 1.0E+092.75E+11 | 4.4E+091.67E+12 | 0.0E+001.62E+11 | 0.0E+001.63E+12 | 2.5E+077.73E+08 | 1.1E+085.71E+09 | 6.5E+072.29E+11 | 2.7E+081.01E+12 | 8.0E+071.07E+11 | 3.4E+086.39E+11 | 1.7E+093.85E+11 | 7.1E+092.00E+12 | 0.0E+005.94E+10 | 0.0E+006.31E+11 | 3.6E+081.03E+10 | 1.5E+098.46E+10 | 5.4E+079.62E+09 | 2.3E+087.20E+10 | 4.4E+085.28E+10 | 1.8E+091.30E+12 | 0.0E+006.75E+09 | 0.0E+002.91E+10 |
| Total | 2.8E+11 | 2.1E+12 | 1.6E+11 | 2.1E+12 | 7.7E+08 | 6.4E+09 | 2.3E+11 | 1.4E+12 | 1.1E+11 | 1.0E+12 | 3.9E+11 | 2.9E+12 | 6.0E+10 | 1.2E+12 | 1.1E+10 | 9.3E+10 | 9.6E+09 | 1.0E+11 | 5.3E+10 | 2.2E+12 | 6.9E+09 | 5.1E+10 |

# Results of comparison of global with Chinese specific results for the BEV and ICEV

Table S 17: Results of comparison of global with Chinese specific results for the BEV and ICEV

|  | Political Stability | | | | Trade barriers | | | |
| --- | --- | --- | --- | --- | --- | --- | --- | --- |
|  | Results global | | Results specific for China | | Results global | | Results specific for China | |
| Resource name | ICEV | BEV | ICEV | BEV | ICEV | BEV | ICEV | BEV |
| Aluminium | 2.83E+09 | 1,50E+10 | 0.00E+00 | 0,00E+00 | 0.00E+00 | 0,00E+00 | 0.00E+00 | 0,00E+00 |
| Cadmium | 8.48E+06 | 8,52E+06 | 1.60E+07 | 1,60E+07 | 0.00E+00 | 0,00E+00 | 0.00E+00 | 0,00E+00 |
| Calcium | 3.52E+06 | 1,55E+08 | 0.00E+00 | 0,00E+00 | 4.47E+06 | 1,97E+08 | 0.00E+00 | 0,00E+00 |
| Chromium | 6.30E+05 | 6,90E+06 | 4.60E+05 | 1,48E+06 | 9.93E+05 | 1,09E+07 | 0.00E+00 | 0,00E+00 |
| Cobalt | 0.00E+00 | 4,83E+11 | 0.00E+00 | 9,30E+10 | 0.00E+00 | 6,65E+11 | 0.00E+00 | 1,30E+11 |
| Crude oil | 0.00E+00 | 0,00E+00 | 5.34E+10 | 9,41E+10 | 0.00E+00 | 0,00E+00 | 5.76E+10 | 1,02E+11 |
| Gold | 2.87E+10 | 2,17E+11 | 0.00E+00 | 0,00E+00 | 4.51E+10 | 3,40E+11 | 0.00E+00 | 0,00E+00 |
| Graphite | 0.00E+00 | 1,94E+11 | 0.00E+00 | 0,00E+00 | 0.00E+00 | 2,28E+11 | 0.00E+00 | 0,00E+00 |
| Lignite coal | 7.39E+07 | 3,94E+09 | 0.00E+00 | 0,00E+00 | 1.07E+08 | 5,71E+09 | 0.00E+00 | 0,00E+00 |
| Iron | 0.00E+00 | 0,00E+00 | 0.00E+00 | 0,00E+00 | 2.49E+09 | 9,07E+10 | 0.00E+00 | 0,00E+00 |
| Lead | 5.12E+09 | 4,12E+09 | 0.00E+00 | 0,00E+00 | 0.00E+00 | 0,00E+00 | 0.00E+00 | 0,00E+00 |
| Magnesium | 4.77E+07 | 2,01E+08 | 0.00E+00 | 0,00E+00 | 5.41E+07 | 2,28E+08 | 0.00E+00 | 0,00E+00 |
| Manganese | 0.00E+00 | 0,00E+00 | 0.00E+00 | 0,00E+00 | 1.06E+09 | 4,39E+09 | 0.00E+00 | 0,00E+00 |
| Nickel | 0.00E+00 | 0,00E+00 | 3.65E+08 | 3,52E+10 | 4.91E+08 | 9,37E+10 | 4.20E+08 | 1,86E+10 |
| Niobium | 1.06E+10 | 1,06E+10 | 1.06E+10 | 2,31E+09 | 2.24E+10 | 2,26E+10 | 1.88E+10 | 4,92E+09 |
| Phosphorus | 1.47E+06 | 1,26E+06 | 0.00E+00 | 0,00E+00 | 1.84E+06 | 1,58E+06 | 0.00E+00 | 0,00E+00 |
| Platinum | 8.17E+10 | 8,62E+10 | 0.00E+00 | 1,84E+10 | 1.46E+11 | 1,54E+11 | 0.00E+00 | 3,30E+10 |
| Silicon | 6.68E+09 | 1,15E+10 | 0.00E+00 | 0,00E+00 | 0.00E+00 | 0,00E+00 | 0.00E+00 | 0,00E+00 |
| Silver | 3.67E+09 | 2,77E+10 | 0.00E+00 | 0,00E+00 | 5.51E+09 | 4,16E+10 | 0.00E+00 | 0,00E+00 |
| Sodium | 0.00E+00 | 0,00E+00 | 0.00E+00 | 0,00E+00 | 0.00E+00 | 7,41E+08 | 0.00E+00 | 0,00E+00 |
| Tantalum | 1.33E+11 | 1,00E+12 | 1.19E+11 | 1,95E+11 | 1.58E+11 | 1,19E+12 | 1.19E+11 | 2,33E+11 |
| Tinn | 1.99E+09 | 8,65E+09 | 0.00E+00 | 0,00E+00 | 2.42E+09 | 1,05E+10 | 0.00E+00 | 0,00E+00 |
| Titanium | 0.00E+00 | 0,00E+00 | 0.00E+00 | 0,00E+00 | 2.89E+08 | 3,74E+08 | 0.00E+00 | 0,00E+00 |
| Zinc | 1.05E+09 | 4,39E+09 | 0.00E+00 | 0,00E+00 | 1.69E+09 | 7,08E+09 | 0.00E+00 | 0,00E+00 |
| Uranium | 0.00E+00 | 9,51E+06 | 0.00E+00 | 8,81E+06 | 0.00E+00 | 1,65E+07 | 0.00E+00 | 0,00E+00 |

# End-of-Life phase results for the ICEV and BEV

It is assumed that the recycling rate of waste steel is 95%, waste non-ferrous metals is 90%, the recycling rate of waste plastics, rubber and glass is 50%. The other parts were modeled according to the waste disposal scenario in CALCD, 20% incinerated and 80% landfill. Due to the wide application of hydrometallurgical methods for recycling waste LIBs in China and in order to simplify our model, it is assumed that used NCM 622 batteries are 100% collected and recycled by hydrometallurgical methods to feed into NCM 622 production loop and thus avoid the production of primary materials, such as steel, aluminum, polypropylene and copper [1].

The End-of-Life phase environmental results for the ICEV and BEV are shown in Table S 18. For all four impact categories, the End-of-Life phase contributions are negative, mainly because of the recycling of waste materials and batteries.

Table S 18 The environmental impacts for the ICEV and BEV in the End-of-Life phase

| Vehilcle type | GWP (t CO_2_ e) | AP (kg SO_2_ e) | POCP (kg C_2_H_4_ e) | EP (kg PO_4_^3-^ e) |
| --- | --- | --- | --- | --- |
| ICEV | -3.4 | -13.1 | -1.8 | -0.9 |
| BEV | -4.1 | -10.2 | -3.1 | -0.9 |

Table S 19 Life cycle environmental impacts comparison for the ICEV and BEV including the End-of-Life phase

| Vehilcle type | Life cyle stage | GWP  (t CO_2_ e) | AP  (kg SO_2_ e) | POCP  (kg C_2_H_4_ e) | EP  (kg PO_4_^3-^ e) |
| --- | --- | --- | --- | --- | --- |
| ICEV | Vehicle cycle | 4.0 | 14.8 | 2.2 | 4.3 |
|  | Fuel cycle | 27 | 16.9 | 2.3 | 1.5 |
|  | Total | 31.0 | 31.7 | 4.5 | 5.8 |
| BEV | Vehicle cycle | 13.7 | 64.5 | 6.0 | 27.8 |
|  | Fuel cycle | 17.8 | 45.2 | 1.8 | 3.6 |
|  | Total | 31.5 | 109.7 | 7.8 | 31.3 |

As shown in Table S 18 and Table S 19, the End-of-Life phase contributions are negative in all the criticality aspects for both the ICEV and BEV, mainly due to the recycling of waste materials and batteries.

Table S 20 The criticality aspects for the ICEV in the End-of-Life phase

| Resource name | Political Stability | Demand growth | Mining capacity | Concentration of reserves | Conecntration of production | Trade barriers | Feasability of exploration projects | Price volatility | Occurence of co-production | Primary material use | Company conecntration |
| --- | --- | --- | --- | --- | --- | --- | --- | --- | --- | --- | --- |
| Aluminium | -2.5E+09 | -1.1E+09 | 0.0E+00 | -1.4E+08 | -6.8E+08 | 0.0E+00 | -1.3E+10 | 0.0E+00 | 0.0E+00 | 0.0E+00 | 0.0E+00 |
| Cadmium | -9.2E+06 | 0.0E+00 | -2.4E+04 | 0.0E+00 | -3.3E+05 | 0.0E+00 | 0.0E+00 | -1.6E+06 | -2.2E+06 | -2.6E+06 | 0.0E+00 |
| Calcium | -3.0E+05 | 0.0E+00 | 0.0E+00 | 0.0E+00 | -1.3E+05 | -3.8E+05 | 0.0E+00 | 0.0E+00 | 0.0E+00 | -1.7E+05 | -2.8E+04 |
| Chromium | -5.7E+05 | -1.3E+05 | -1.6E+04 | -1.7E+05 | -9.1E+04 | -8.9E+05 | -2.3E+06 | 0.0E+00 | 0.0E+00 | -2.2E+05 | 0.0E+00 |
| Copper | 0.0E+00 | 0.0E+00 | 0.0E+00 | 0.0E+00 | 0.0E+00 | 0.0E+00 | 0.0E+00 | 0.0E+00 | 0.0E+00 | 0.0E+00 | 0.0E+00 |
| Crude oil | 0.0E+00 | 0.0E+00 | -4.8E+06 | -6.5E+07 | 0.0E+00 | 0.0E+00 | 0.0E+00 | 0.0E+00 | -5.2E+07 | -3.5E+08 | 0.0E+00 |
| Gold | 0.0E+00 | 0.0E+00 | -7.6E+04 | 0.0E+00 | 0.0E+00 | 0.0E+00 | -1.7E+08 | -5.4E+07 | 0.0E+00 | -2.0E+07 | -6.0E+05 |
| Iron | -1.4E+10 | 0.0E+00 | -2.5E+08 | 0.0E+00 | 0.0E+00 | -2.3E+10 | 0.0E+00 | 0.0E+00 | 0.0E+00 | -4.4E+09 | 0.0E+00 |
| Lead | 0.0E+00 | -2.5E+08 | -5.0E+06 | -2.6E+07 | -7.6E+07 | -7.7E+08 | 0.0E+00 | -4.0E+08 | 0.0E+00 | 0.0E+00 | 0.0E+00 |
| Magnesium | -4.5E+09 | 0.0E+00 | -9.9E+07 | -5.0E+08 | -8.3E+08 | 0.0E+00 | 0.0E+00 | -1.7E+09 | -2.4E+08 | 0.0E+00 | 0.0E+00 |
| Manganese | -4.3E+07 | -1.2E+08 | 0.0E+00 | -2.1E+06 | -2.3E+07 | -4.9E+07 | 0.0E+00 | 0.0E+00 | -1.5E+06 | 0.0E+00 | -8.4E+05 |
| Nickel | 0.0E+00 | -1.6E+08 | -2.6E+06 | -4.6E+07 | -5.4E+07 | -1.0E+09 | -2.5E+09 | 0.0E+00 | 0.0E+00 | 0.0E+00 | 0.0E+00 |
| Niobium | 0.0E+00 | -2.4E+08 | -1.1E+06 | 0.0E+00 | 0.0E+00 | -4.3E+08 | 0.0E+00 | -8.0E+07 | -1.2E+07 | 0.0E+00 | 0.0E+00 |
| Phosphorus | -1.0E+10 | 0.0E+00 | 0.0E+00 | -1.8E+10 | -1.7E+10 | -2.2E+10 | 0.0E+00 | 0.0E+00 | -4.8E+08 | -4.0E+09 | -4.1E+09 |
| Platinum | -1.4E+06 | -3.1E+05 | 0.0E+00 | -6.7E+05 | -2.7E+05 | -1.8E+06 | -1.1E+07 | -4.8E+05 | 0.0E+00 | -6.8E+05 | -1.2E+05 |
| Silicon | -6.9E+10 | -1.7E+10 | 0.0E+00 | -1.2E+11 | -5.7E+10 | -1.2E+11 | 0.0E+00 | 0.0E+00 | -3.5E+09 | 0.0E+00 | -1.5E+09 |
| Silver | -4.7E+09 | 0.0E+00 | 0.0E+00 | -1.8E+09 | -2.1E+09 | 0.0E+00 | -1.6E+10 | 0.0E+00 | 0.0E+00 | 0.0E+00 | -4.8E+08 |
| Tantalum | -1.8E+09 | 0.0E+00 | -2.6E+07 | 0.0E+00 | 0.0E+00 | -2.8E+09 | 0.0E+00 | 0.0E+00 | -3.5E+08 | -6.2E+08 | 0.0E+00 |
| Tin | -6.6E+10 | -6.9E+10 | 0.0E+00 | -3.1E+10 | -7.7E+09 | -7.9E+10 | 0.0E+00 | 0.0E+00 | -1.9E+09 | -1.6E+10 | 0.0E+00 |
| Titanium | -1.5E+09 | 0.0E+00 | -3.2E+07 | 0.0E+00 | -1.3E+08 | -1.9E+09 | 0.0E+00 | 0.0E+00 | 0.0E+00 | -3.8E+08 | 0.0E+00 |
| Zinc | -9.42E+08 | 0.00E+00 | -2.27E+07 | -5.84E+07 | -7.24E+07 | -1.52E+09 | 0.00E+00 | -3.26E+08 | -4.88E+07 | -3.95E+08 | 0.00E+00 |

Table S 21 The criticality aspects for the BEV in the End-of-Life phase

| Resource name | Political Stability | Demand growth | Mining capacity | Concentration of reserves | Conecntration of production | Trade barriers | Feasability of exploration projects | Price volatility | Occurence of co-production | Primary material use | Company conecntration |
| --- | --- | --- | --- | --- | --- | --- | --- | --- | --- | --- | --- |
| Aluminium | -1.1E+10 | -4.8E+09 | 0.0E+00 | -6.4E+08 | -3.1E+09 | 0.0E+00 | -5.7E+10 | 0.0E+00 | 0.0E+00 | 0.0E+00 | 0.0E+00 |
| Cadmium | -7.7E+06 | 0.0E+00 | -2.0E+04 | 0.0E+00 | -2.7E+05 | 0.0E+00 | 0.0E+00 | -1.3E+06 | -1.8E+06 | -2.1E+06 | 0.0E+00 |
| Calcium | -4.0E+05 | 0.0E+00 | 0.0E+00 | 0.0E+00 | -1.7E+05 | -5.1E+05 | 0.0E+00 | 0.0E+00 | 0.0E+00 | -2.3E+05 | -3.8E+04 |
| Chromium | -6.2E+06 | -1.4E+06 | -1.8E+05 | -1.9E+06 | -9.9E+05 | -9.8E+06 | -2.5E+07 | 0.0E+00 | 0.0E+00 | -2.4E+06 | 0.0E+00 |
| Copper | -1.4E+11 | -4.0E+10 | -2.2E+08 | -1.3E+10 | -2.2E+10 | -2.0E+11 | -3.1E+11 | 0.0E+00 | -1.5E+10 | -2.3E+10 | 0.0E+00 |
| Crude oil | 0.0E+00 | 0.0E+00 | -2.4E+07 | -3.3E+08 | 0.0E+00 | 0.0E+00 | 0.0E+00 | 0.0E+00 | -2.6E+08 | -1.8E+09 | 0.0E+00 |
| Gold | 0.0E+00 | 0.0E+00 | -1.1E+05 | 0.0E+00 | 0.0E+00 | 0.0E+00 | -2.5E+08 | -7.9E+07 | 0.0E+00 | -2.9E+07 | -8.8E+05 |
| Iron | -1.1E+11 | 0.0E+00 | -1.9E+09 | 0.0E+00 | 0.0E+00 | -1.7E+11 | 0.0E+00 | 0.0E+00 | 0.0E+00 | -3.3E+10 | 0.0E+00 |
| Lead | 0.0E+00 | -2.3E+08 | -4.6E+06 | -2.4E+07 | -7.0E+07 | -7.1E+08 | 0.0E+00 | -3.7E+08 | 0.0E+00 | 0.0E+00 | 0.0E+00 |
| Magnesium | -3.6E+09 | 0.0E+00 | -7.8E+07 | -4.0E+08 | -6.5E+08 | 0.0E+00 | 0.0E+00 | -1.3E+09 | -1.9E+08 | 0.0E+00 | 0.0E+00 |
| Manganese | -1.8E+08 | -5.0E+08 | 0.0E+00 | -8.7E+06 | -9.7E+07 | -2.1E+08 | 0.0E+00 | 0.0E+00 | -6.2E+06 | 0.0E+00 | -3.6E+06 |
| Nickel | 0.0E+00 | -3.2E+08 | -5.1E+06 | -9.1E+07 | -1.1E+08 | -2.1E+09 | -5.0E+09 | 0.0E+00 | 0.0E+00 | 0.0E+00 | 0.0E+00 |
| Niobium | 0.0E+00 | -1.6E+10 | -7.5E+07 | 0.0E+00 | 0.0E+00 | -2.9E+10 | 0.0E+00 | -5.4E+09 | -8.0E+08 | 0.0E+00 | 0.0E+00 |
| Phosphorus | -1.0E+10 | 0.0E+00 | 0.0E+00 | -1.8E+10 | -1.7E+10 | -2.2E+10 | 0.0E+00 | 0.0E+00 | -4.7E+08 | -3.9E+09 | -4.1E+09 |
| Platinum | -1.2E+06 | -2.6E+05 | 0.0E+00 | -5.6E+05 | -2.3E+05 | -1.5E+06 | -9.5E+06 | -4.1E+05 | 0.0E+00 | -5.7E+05 | -1.0E+05 |
| Silicon | -4.3E+10 | -1.1E+10 | 0.0E+00 | -7.7E+10 | -3.6E+10 | -7.7E+10 | 0.0E+00 | 0.0E+00 | -2.2E+09 | 0.0E+00 | -9.3E+08 |
| Silver | -8.5E+09 | 0.0E+00 | 0.0E+00 | -3.3E+09 | -3.8E+09 | 0.0E+00 | -2.8E+10 | 0.0E+00 | 0.0E+00 | 0.0E+00 | -8.7E+08 |
| Tantalum | -1.4E+10 | 0.0E+00 | -2.0E+08 | 0.0E+00 | 0.0E+00 | -2.1E+10 | 0.0E+00 | 0.0E+00 | -2.7E+09 | -4.7E+09 | 0.0E+00 |
| Tin | -5.0E+11 | -5.2E+11 | 0.0E+00 | -2.3E+11 | -5.8E+10 | -6.0E+11 | 0.0E+00 | 0.0E+00 | -1.4E+10 | -1.2E+11 | 0.0E+00 |
| Titanium | -5.8E+09 | 0.0E+00 | -1.2E+08 | 0.0E+00 | -4.8E+08 | -7.0E+09 | 0.0E+00 | 0.0E+00 | 0.0E+00 | -1.4E+09 | 0.0E+00 |
| Zinc | 0.0E+00 | 0.0E+00 | 0.0E+00 | -1.0E+07 | 0.0E+00 | -3.2E+08 | 0.0E+00 | -2.0E+08 | 0.0E+00 | 0.0E+00 | 0.0E+00 |

# References

[1] Sun, X., et al., Life cycle assessment of lithium nickel cobalt manganese oxide (NCM) batteries for electric passenger vehicles. Journal of Cleaner Production, 2020. **273**: p. 123006.
